# Natural Ageing-Related Alterations of Biological Markers in Maize Seeds Under Ex-Situ Conservation

**DOI:** 10.3390/ijms262412124

**Published:** 2025-12-17

**Authors:** Natalija Kravic, Sladjana Zilic, Jelena Vukadinovic, Tanja Petrovic, Marija Milivojevic, Jelena Srdic, Marijana Simic, Snezana Mladenovic Drinic, Violeta Andjelkovic

**Affiliations:** Maize Research Institute Zemun Polje, Slobodana Bajica 1, 11185 Belgrade, Serbia; szilic@mrizp.rs (S.Z.); mesarovicj16@gmail.com (J.V.); ptanja@mrizp.rs (T.P.); mmarija@mrizp.rs (M.M.); jsrdic@mrizp.rs (J.S.); marijana.simic@mrizp.rs (M.S.); drinicsnezana2@gmail.com (S.M.D.); violeta@mrizp.rs (V.A.)

**Keywords:** carotenoids, cold storage, early seedling growth, phenolics, proteins, seed viability, sugars, tocopherols, *Zea mays* L.

## Abstract

Contemporary seed gene bank management emphasizes the importance of understanding seed storage behaviour to maximize the preservation of genetic material. In this context, the patterns of naturally occurring ageing-related changes in physiological and biochemical markers were evaluated by comparing the performance of freshly regenerated seed samples (control) to samples kept under cold storage (CS) for 37 years (original, CS1 samples) and five years (CS2 samples). A significant decline in seed viability and physiological quality—initial seedling development—was directly associated with the duration of cold storage, leading to a marked reduction in seedling vigour index (SVI) performance. Key biochemical markers influencing early seedling growth and vigour included total protein, the glutelins protein fraction, fructose, sucrose, both insoluble-bound and soluble-free phenolics—including phenolic acids, and *β*-carotene. The CS2 samples, which experienced severe water deficit during the post-zygotic phase, exhibited increased sucrose, insoluble-bound *p*-coumaric acid (*p*-CouA), insoluble-bound ferulic acid (FA), and *α*-tocopherol contents. Conversely, glutelins and glucose contents decreased, while genotype-specific variations were observed in albumins, globulins, fructose, maltose, insoluble-bound caffeic acid, and soluble-free *p*-CouA, as well as in *β+γ*- and *δ*-tocopherol contents. Given the consistent pattern of natural ageing-related changes, *β*-carotene, lutein+zeaxanthin, insoluble-bound FA, and particularly soluble-free FA emerge as relevant biomarkers for improved monitoring of ageing processes under ex situ conservation.

## 1. Introduction

The ex situ conservation of seed collections represents a vital strategy for both the current and future utilization of plant genetic resources [1]. In this context, seed viability emerges as a crucial factor in the effective management of these collections. Since the physiological and physical state of seeds directly influences their storage behaviour, it is imperative that seed gene banks provide optimal storage conditions to uphold genetic integrity and minimize viability loss [2,3].

During the maturation phase, a late stage of embryogenesis, orthodox seeds undergo desiccation, resulting in the release of most tissue water and reaching equilibrium with the relative humidity of the surrounding environment [4]. Prior to or in parallel to maturation, orthodox seeds acquire desiccation tolerance through a series of protective processes and highly efficient biological mechanisms, which include (1) the maintenance of cellular integrity in a dehydrated state; (2) intracellular dedifferentiation marked by the endomembrane reduction and a diminishing contribution of mitochondria and plastids to the cell’s cross-sectional area as development progresses; (3) the capacity for vitrification at low water potential; (4) the ability to modulate metabolism, effectively conserving energy via a significant reduction in respiration rates as development progresses; (5) the control of reactive oxygen species (ROS) generated during desiccation through antioxidant pools; and (6) the activation of protective metabolic changes, particularly the metabolism of specific proteins and carbohydrates [5,6,7]. As a result, the capacity to tolerate desiccation and remain dry and quiescent for extended periods enhances both the storage potential and viability of orthodox seeds under the medium- and long-term storage conditions typically employed in gene banks [8,9].

As seeds undergo maturation, they gradually acquire longevity, defined as the total timespan during which desiccated seeds remain viable. In seeds classified as orthodox [10], seed viability (i.e., germination ability) and quality (i.e., vigour) are regulated by three critical factors: seed moisture content, which must be in equilibrium with the relative humidity (ERH) of the surrounding atmosphere; storage temperatures; and the gaseous environment [11,12,13,14]. Beyond the conditions of storage, it is very important to consider how the environmental factors experienced by the maternal plant during the post-zygotic phase influence seed longevity [15,16,17]. Existing research indicates that the climate of origin significantly contributes to the considerable variability in seed longevity observed across different populations of the same species [17,18]. Therefore, understanding seed longevity is particularly relevant for crops like maize, which exhibit a range of adaptations to local environments and climatic conditions across their diverse landraces [19]. However, the longevity of orthodox seeds is primarily attributed to genetic makeup influencing key internal seed features (structure and chemical composition) as well as germination phenology and is upregulated by an array of molecular and physiological factors that govern protective and reparative mechanisms [11,18,20,21,22].

Even under optimal conditions, ageing and the subsequent onset of degradation processes can lead to a loss of seed viability. Seed ageing is an irreversible, cumulative, and inevitable phenomenon, resulting in the accumulation of cellular damage [23]. This damage can impede seedling emergence and heighten susceptibility to environmental stressors during both the germination phase and the early stages of plant growth. Seeds do not exhibit uniform ageing; even within a single seed lot, individual seeds display differing potentials for storage viability. During the initial imbibition phase, the limited capacity for cellular repair—an energy- and time-intensive process—can delay the average germination rate, particularly with respect to radicle protrusion [24]. In cases where seeds have undergone substantial deterioration, successful radicle protrusion may result in the formation of abnormal seedlings. Conversely, seeds that have experienced extreme degradation exhibit a complete absence of radicle protrusion, ultimately disabling seedling formation [6].

Seed ageing is a complex biological process that involves a multitude of interconnected biochemical, molecular, metabolic, and physiological mechanisms [24,25]. These mechanisms include lipid peroxidation [26], protein carbonylation [27], the Amadori–Maillard reaction [28], and programmed cell death [29]. The natural ageing process, which occurs during extended storage in cold and dry environments or through accelerated ageing under adverse conditions (i.e., elevated temperature and humidity) [30], disrupts the delicate balance between the generation of reactive oxygen species and the functional capacity of antioxidant defence systems and pools [31]. However, the validity of extrapolating seed longevity estimates derived from accelerated ageing experiments performed under adverse conditions to gene banks’ storage conditions is questionable, as the underlying deterioration mechanisms may differ [32].

To date, only a few studies have compared the lifespan of seeds across accessions of a single plant species [33,34,35]. Furthermore, very few studies on seed longevity have been performed with long-term-stored seed material [11,33,36]. The Maize Research Institute Zemun Polje (MRIZP) gene bank hosts an impressive active collection of nearly 6000 accessions, positioning it among the ten largest maize gene banks globally [37]. In a recent survey of orange-coloured gene bank’s accessions, we noted significant variations in colour intensity between the original seed lots harvested in 1985 and the same ones regenerated in 2017. Due to a severe water shortage in 2017, the original seed lots underwent repeated regeneration in 2022.

To ensure the long-term viability and quality of ex situ seed collections, it is essential to understand the differential ageing rates across various accessions. Hence, this study aims to enhance our knowledge of seed decay mechanisms, with a particular focus on key factors that have not been comprehensively examined. Our primary objectives are (1) to evaluate the impact of environmental conditions during the regeneration year on seed lots’ performance and seedling growth; (2) to analyse the natural changes in physiological and biochemical markers associated with ageing during cold storage over a period of up to 37 years; and (3) to identify the most reliable biochemical markers that can predict or estimate seed viability loss, thereby supplementing standard germination tests.

## 2. Results

The pattern of natural ageing-related change in physiological and biochemical markers during medium-term cold storage (CS) was systematically evaluated. This assessment involved a comparative analysis of freshly regenerated seed samples that were harvested in 2022 and served as the control group against two distinct groups of samples subjected to varying durations of cold storage: the original CS1 samples, harvested in 1985, which had undergone a 37-year cold storage period, and the CS2 samples, harvested in 2017, which had been stored for five years.

### 2.1. Seed Germination and Seedling Vigour

Seeds’ ability to germinate exhibited a clear pattern of variation in response to the natural ageing process. The initial germination percentage of the original CS1 samples exceeded 98%. However, after 37 years of cold storage, there was a significant average decline of approximately 78% in seed germinability. In contrast, samples with a five-year cold storage time span preserved a high germination percentage of over 98%.

Furthermore, a notable trend associated with seed ageing was the overall deterioration in the performance of all evaluated seedling growth parameters. Specifically, a reduction in seedling root fresh weight (FW) was recorded, with an average decline of 83% for CS1 samples and 8% for CS2 samples. This was followed by a decrease in seedling shoot FW, which averaged 78% for CS1 samples and approximately 10% for CS2 samples, alongside reductions in seed residue FW, averaging around 58% for CS1 and 10% for CS2. A similar pattern and magnitude of decline were observed in seedling root, shoot, and seed residue dry weight (DW) for both CS1 and CS2 samples. Additionally, a substantial decrease in both root and shoot lengths was evidenced in the original CS1 samples, with reductions of approximately 54% and 51%, respectively. However, the CS2 samples expressed only a slight 3% decrease in these parameters (Table 1).

**Figure 1:**
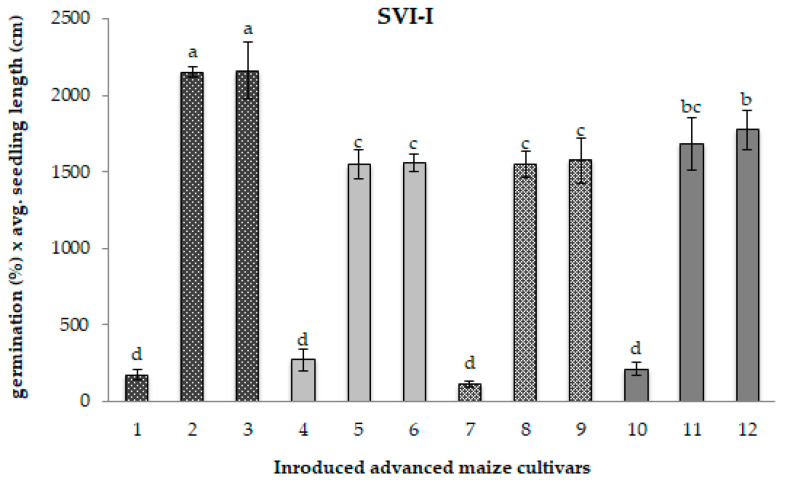
Seedling Vigour Index-I for 12 maize samples differing in the period of maintenance under cold storage. Different chart bars followed by the same letter are not significantly different (*p* ≤ 0.05). Samples 1, 4, 7, and 10 harvested in 1985 (CS1); samples 2, 5, 8, and 11 regenerated in 2017 (CS2); samples 3, 6, 9, and 12 freshly regenerated in 2022 (control). The seed samples belong to the following maize genotypes: MRIZP13509 (samples 1, 2, and 3); MRIZP13695 (samples 4, 5, and 6); MRIZP13687 (samples 7, 8, and 9); MRIZP13706 (samples 10, 11, and 12).

**Figure 2:**
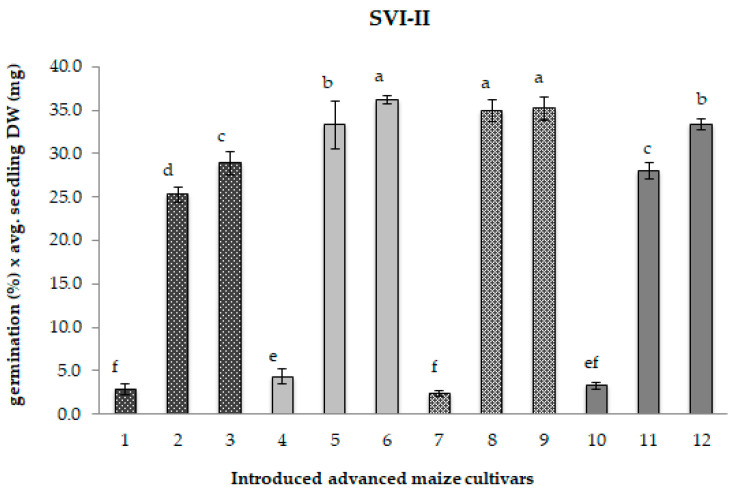
Seedling Vigour Index-II for 12 maize samples differing in the period of maintenance under cold storage. Different chart bars followed by the same letter are not significantly different (*p* ≤ 0.05). Samples 1, 4, 7, and 10 harvested in 1985 (CS1); samples 2, 5, 8, and 11 regenerated in 2017 (CS2); samples 3, 6, 9, and 12, freshly regenerated in 2022 (control). The seed samples belong to the following maize genotypes: MRIZP13509 (samples 1, 2, and 3); MRIZP13695 (samples 4, 5, and 6); MRIZP13687 (samples 7, 8, and 9); MRIZP13706 (samples 10, 11, and 12).

As a consequence of an extended period of cold storage, the significant decline in both the germination percentage and seedling growth parameters led to a considerable reduction in the Seedling Vigour Indices (SVI-I and SVI-II), relevant indicators of seed performance potential, both under field conditions and during storage, as illustrated in Figure 1 and Figure 2.

Compared to the matching control samples, the original CS1 samples exhibited a highly pronounced average reduction of 90% in both the SVI-I—which includes germination percentage and total seedling length—and the SVI-II, which considers germination percentage and total seedling dry weight (Figure 1 and Figure 2). In contrast, the CS2 samples displayed only a slight decline in seedling vigour, with reductions of approximately 2% for SVI-I and around 9% for SVI-II.

### 2.2. Biochemical Markers

#### 2.2.1. Analysis of Total Protein and Protein Fractions

The analysis revealed that total protein content remained unchanged during natural seed ageing (Table 2). In comparison to controls, the original CS1 samples displayed a slight average decrease in total protein content of approximately 3%. Conversely, when compared to the matching controls, the CS2 samples displayed a slight average increase in total protein content of nearly 3% (Table 2).

However, the analysis revealed a significant, although inconsistent, pattern of variation in the individual protein fractions’ content associated with seed ageing (Table 2). Notably, concerning the protein fractions’ content, the most pronounced decrease was observed for albumins (i.e., a 38% decrease), followed by a ~7% decline in the α-zeins. The majority of the CS1 samples expressed an average decline in the globulins fraction of ~5% and an average increase in the glutelins fraction of 4.5%; however, the exception was sample 7, which displayed the opposite trend of change for the given protein fractions (i.e., a ~1% increase in globulins and a ~5% decrease in glutelins, respectively).

The majority of the CS2 samples expressed an average decline in protein fractions: specifically, reductions of approximately 16%, 11%, 10%, and 3% in albumins, glutelins, α-zeins, and globulin fractions, respectively. However, exceptions were noted in samples 11 and 2, which displayed opposite trends of change for the given protein fractions: sample 11 showed increases of ~9%, 1%, and 3% in albumins, globulins, and α-zeins, respectively, while sample 2 exhibited a ~10% increase in glutelins (Table 2).

#### 2.2.2. Analysis of Reducing and Non-Reducing Sugars

In comparison to the performance of control samples, the majority of the original CS1 samples, as well as the CS2 samples, exhibited a significant average reduction in glucose (~19%, i.e., 17%) and particularly maltose (~26%, i.e., 21%) content. The opposite upward trend of change for the given sugars was observed in CS1 sample 10 (i.e., 11% increase in glucose content) and in CS2 sample 11 (i.e., 12% increase in maltose content), respectively (Table 3).

The majority of the original CS1 samples exhibited an average decrease of approximately 6% in sucrose content, a non-reducing sugar, with the exception of sample 1, which displayed a notable increase of 42%. Additionally, an average increase in sucrose content of ~13% was observed in all CS2 samples (e.g., samples regenerated under conditions of extreme water scarcity), with sample 2 exhibiting the most significant increase of ~23%. Both CS1 sample 1 and CS2 sample 2 belong to the genotype that differs in origin from the other three advanced maize cultivars introduced (Table 3).

The natural ageing-related alterations in fructose content were found to be the most pronounced. When compared to the performance of control samples, the majority of CS1 samples displayed a significant average increase in fructose content of 68%, with samples 10 and 7 revealing an increase of approximately 74% and 114%, respectively. The CS2 samples exhibited genotype-specific variations in fructose content changes compared to the control samples: samples 5 and 8 showed an average increase of about 31% in fructose content, while samples 2 and 11 expressed an average decrease of approximately 33%, respectively (Table 3).

#### 2.2.3. Analysis of Soluble-Free and Insoluble-Bound Phenolics

The analysis revealed that the majority of CS1 samples exhibited similar levels of total soluble-free phenolics (FPs) and insoluble-bound phenolics (IBPs) compared to their respective controls. However, CS1 sample 7 showed a 13.7% increase in total FPs, while CS1 sample 10 had a 9% increase in total IBPs (Table 4a,b). In contrast, most CS2 samples maintained unchanged total IBPs contents, except for CS2 sample 2, which showed a 10% increase. Regarding FPs, CS2 samples displayed genotype-specific variations: samples 2 and 11 showed no significant change, whereas samples 5 and 9 exhibited increases of 12% and 13%, respectively, compared to controls (Table 4a,b).

The absence of detectable soluble-free caffeic acid (CA) in most of the maize seed lots evaluated precluded an assessment of the potential temporal changes in its content during cold storage. In contrast, CS1 samples expressed a significant average increase of 72% in the soluble-free *p*-coumaric acid (*p*-CouA) content, with values ranging from a 20% increase in CS1 sample 4 to a striking 139% increase in CS1 sample 1. The CS2 samples, however, exhibited more intricate variations in *p*-CouA content change: samples 8 and 11 displayed an average increase of 23%, whereas samples 2 and 5 expressed an average decrease of 20%. Notably, soluble-free ferulic acid (FA) consistently displayed a pronounced upward trend in correlation with the seed ageing process (Table 4a,b).

The majority of CS1 samples exhibited an average decline of approximately 18% in insoluble-bound *p*-CouA content compared to corresponding controls. In contrast, the majority of CS2 samples expressed an average increase of about 18%. However, two exceptions to this trend were observed: CS1 sample 1, which displayed a *p*-CouA content increase of approximately 15%, and CS2 sample 11, which showed a decrease of about 15%. These inconsistencies imply that the observed variations do not stem from ageing-related processes but rather reflect genotype-specific alterations (Table 4a,b).

Conversely, the results revealed a clear pattern wherein the increase in insoluble-bound FA content correlates with a reduction in the duration of cold storage (Table 4a,b).

The majority of CS1 and CS2 samples expressed an average decrease of 14%, i.e., 8% in insoluble-bound CA content when compared to matching controls, indicating that a decreased bound-form CA is largely a pattern of ageing-related change coinciding with reduced cold storage duration. However, two exceptions were noted: CS1 sample 10 and CS2 sample 5, which exhibited increased bound-form CA content by 15% and 17%, respectively, implying a genotype-specific alteration (Table 4a,b).

#### 2.2.4. Analysis of Tocopherols and Carotenoids

The original CS1 samples expressed a clear pattern of change in tocopherol (T) content following an extended 37-year period of cold storage. Specifically, these samples exhibited average reductions in *α*-, *β+ϒ*-, and *δ*-T content of 28%, 25%, and 52%, respectively, in comparison to matching controls. Furthermore, most of the CS2 samples showed a modest average decline of 7.6% in *β+ϒ*-T; however, exceptions were observed in CS1 sample 7 and CS2 sample 5, which both expressed increases of 11% in *δ*-T and 16% in *β+ϒ*-T. Conversely, the majority of CS2 samples displayed an average increase of 22.5% in *α*-T, with variations ranging from 4.3% in sample 11 to 56.4% in sample 5. Notably, the CS2 samples displayed more complex patterns of *δ*-T content variation: samples 2 and 5 showed an average decrease of 25.4%, while samples 8 and 11 showed an average increase of 14.4%. This pattern of variation suggests a genotype-specific type of change rather than an aging-related response (Table 5).

Regarding the carotenoids content, CS1 samples exhibited a significant average decrease of 88.8% in *β*-carotene and a 74.9% reduction in lutein+zeaxanthin compared to their corresponding controls. The CS2 samples displayed similar, although less pronounced, changes in the given phytochemicals’ content (i.e., 38% and 40% decrease, respectively). These findings strongly indicated a clear pattern of change (i.e., a trend of content decrease) in parallel with seed ageing (Table 5).

#### 2.2.5. Analysis of the Total Antioxidant Capacity

**Figure 3:**
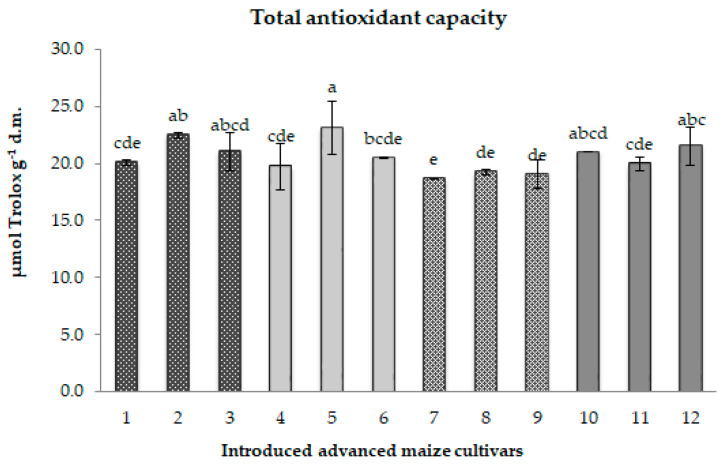
Total antioxidant capacity for 12 maize samples differing in the period of maintenance under cold storage. Different chart bars followed by the same letter are not significantly different (*p* ≤ 0.05). Samples 1, 4, 7, and 10 harvested in 1985 (CS1); samples 2, 5, 8, and 11 regenerated in 2017 (CS2); samples 3, 6, 9, and 12, freshly regenerated in 2022 (control). The seed samples belong to the following maize genotypes: MRIZP13509 (samples 1, 2, and 3); MRIZP13695 (samples 4, 5, and 6); MRIZP13687 (samples 7, 8, and 9); MRIZP13706 (samples 10, 11, and 12).

According to the analysis, the duration of the cold storage period did not have a significant impact on the total antioxidant capacity (TAC). In comparison to their matching control groups, CS1 and CS2 samples displayed contrasting trends in the TAC change. Specifically, the original CS1 samples exhibited a slight average TAC decrease of approximately 3%. Conversely, the majority of CS2 samples expressed an average TAC increase of 7%; the exception was CS2 sample 11, showing a TAC decline of 7% (Figure 3).

### 2.3. Correlations

To evaluate the effects of cold storage duration and environmental conditions during the post-zygotic phase on seed storage behaviour, we analysed the relationship between seed viability, i.e., physiological quality traits, and biochemical markers. This analysis was conducted separately for the CS1, CS2, and non-aged control seed lots. We only presented correlations that were statistically significant and occurred simultaneously in both the control group and at least one of the aged seed groups (CS1 or CS2) (Table 6). In that context, it has been shown that non-aged control seeds and CS2 seeds most frequently exhibited the same trend in the correlations between seedling root length, i.e., SVI-I (which includes percentage of germination and total seedling length), i.e., SVI-II (which includes percentage of germination and total seedling dry weight), and the biochemical markers presented, although the statistical significance of these correlations varied. Additionally, CS1 and CS2 seeds most frequently displayed the same trend in correlations between seedling shoot length and the biochemical markers.

The most consistent trend in correlations across all three seed lot groups was observed between seedling shoot length, i.e., SVI-II, and the majority of the biochemical markers presented, with the exception of fructose and sucrose. The key biochemical markers influencing early seedling growth and SVI performance included total protein; the glutelins protein fraction, fructose, sucrose, both insoluble-bound and soluble-free phenolics—including phenolic acids, and *β+ϒ*-tocopherols, as well as *β*-carotene. The correlation analysis indicated that overall seedling performance is strongly influenced by the specific form of phenolic compounds—whether insoluble-bound or soluble-free (Table 6).

## 3. Discussion

### 3.1. Seed Germination and Seedling Vigour

Over time, stored seeds naturally undergo deterioration due to physical, physiological, and biochemical changes. These processes reduce their viability, vigour, and lifespan [38], directly impacting germination success. Therefore, enhancing seed longevity is crucial for effective long-term storage (germplasm conservation) and crop improvement [39].

Building on this foundation, research consistently demonstrates that long-term storage—spanning multiple decades—significantly reduces seed viability and physiological quality across various species, regardless of their inherent longevity. Additionally, the relationship between seed moisture content and equilibrium relative humidity (ERH) varies depending on storage conditions—whether in active or base collections [33]. These differences significantly influence seed vitality, highlighting the complex interplay of storage environment parameters and seed longevity.

For instance, studies on white clover (*Trifolium repens*), a longer-lived perennial, and red clover (*Trifolium pratense*), a shorter-lived perennial, revealed that storing seeds for up to 40 years in dark, dry, and cool conditions (15–20 °C) leads to significant declines in germinability and seedling growth compared to fresh controls [40]. Similar declines were observed in alfalfa (*Medicago sativa* L.), another long-lived perennial [41]. Even under ultra-dried storage conditions—maintaining seed moisture at 3–5% and temperature at −3.5 ± 0.2 °C over 30 years—seeds still experienced viability declines, with median germination dropping to approximately 80%. Rye (*Secale cereale* L.), with medium-short seed longevity, experienced the most pronounced decline, with viability decreasing by up to 49% [42]. These findings underscore that, even under optimal storage conditions, seed deterioration over extended periods remains unavoidable.

In our study, we examined seed samples from improved maize cultivars stored for up to 37 years in an active collection. Extended storage increased susceptibility to ageing, impairing seed viability and physiological quality, which negatively affected germination and seedling vigour [9]. To assess these effects, we measured key physiological traits—germination percentage and seedling growth parameters (length, fresh weight, and dry weight)—as reliable indicators of seed vitality and potential for successful crop establishment [43].

We observed a significant decline—approximately 78% in germination capacity and around 90% in seedling vigour indices—highlighting the detrimental impact of prolonged cold storage. These reductions indicate impaired capacity for seedling establishment, potentially limiting nutrient uptake and overall plant performance. Our findings align with existing knowledge, suggesting that ageing induces oxidative stress, resulting in cellular damage and a subsequent decrease in metabolic activity essential for seedling growth and development [44,45,46].

Interestingly, while SVI-I values after five years of storage resembled those of freshly regenerated control samples, overall seed quality exhibited signs of deterioration. This was evidenced by alterations in the Seedling Vigour Index-II (SVI-II), observable in the majority of CS2 samples. The extreme water deprivation during the post-zygotic phase in the 2017 regeneration season likely impaired seed development, leading to reductions in seed dry weight and overall seed quality [47]. Our findings corroborate previous reports that water deficits during seed maturation reduce seed size and impose physiological and biochemical changes [48], which, in turn, may further compromise seed viability during storage. The notable exception was sample 8, which maintained stability in SVI-II, which sustained response to storage—characterized by stable SVI-II values—should primarily be attributed to intrinsic genetic factors—seed structural and chemical composition [49], as well as seed heteromorphy [22]. 

Despite these insights, understanding seed longevity remains a complex challenge. A broad-scale study analysing 42,000 seed accessions across 276 species—assessing seed longevity after storage at 5 °C and, for a subset, at −18 °C—revealed that the time for germination to decline by 50% (P_50_) varies widely—from under 13 years to over 450 years—with a median of approximately 54 years [20]. This variability underscores that, although progress has been made, our comprehension of the traits that govern seed storage behaviour is still evolving. Moreover, meta-analyses comparing predicted germinability with experimental observations often find that models tend to overestimate actual viability, emphasizing the need for more accurate, reliable markers—whether physiological, biochemical, or molecular—to improve longevity predictions during storage [3,12,50].

### 3.2. Biochemical Markers

#### 3.2.1. Analysis of Total Protein and Protein Fractions

Seeds stored in gene banks under cold conditions gradually age due to a continued accumulation of free radicals [51]. At low seed moisture content, protection against oxidative damage is more likely to rely on non-enzymatic ROS scavenging systems—primarily seed storage proteins (SSPs)—rather than enzymatic detoxification mechanisms [52,53]. The abundance of these non-enzymatic ROS scavengers, mainly SSPs, is regulated during seed development and maturation and can be influenced by environmental conditions during the post-zygotic phase [54]. The SSPs are considered primary targets for oxidation in seeds [55]. Their high abundance and affinity for oxidation suggest that SSPs serve as an effective ROS buffering system, protecting cellular components essential for embryo survival. This protective role has been linked to a chaperone-like function of SSPs during seed dry storage [56].

Soluble proteins in seeds comprise a class of functional proteins necessary to maintain cell survival—they supply nitrogen, sulfur, carbon, and essential amino acids for seed germination and seedling growth [57]. During seed aging, storage proteins typically undergo gradual changes, with more pronounced ageing leading to a decrease in SSPs content [58,59]. Interestingly, our analysis of total protein and individual protein fractions revealed no consistent pattern linked to seed ageing. Notably, in CS1 samples, we observed a uniform decline in α-zeins and albumins, along with a predominant decrease in total protein and globulins, followed by genotype-specific shifts in glutelins content compared to controls. Conversely, CS2 samples exhibited a predominant decrease in glutelins but an increase in total protein, followed by genotype-specific variations in albumins, globulins, and *α*-zeins relative to controls.

The main cause of protein reduction during natural seed ageing is oxidative damage from reactive oxygen species. Thus, regarding a decrease in seed total protein and individual protein fractions content during natural ageing, it has been shown that excessive ROS induces protein oxidation (i.e., carbonylation), which, despite being non-enzymatic, can affect the enzymes’ activities and increase the susceptibility of proteins to proteolysis, which is associated with seed deterioration in many crops [60]. However, the loss of seed vigour induced by ageing is likely not caused by the lack of protein reserves but is the result of protein changes (i.e., impaired protein function) in dry CS1 seeds during prolonged cold storage, the regulation of protein synthesis and turnover, posttranslational modifications (PTMs), and a reduction in translational activity during germination [61]. Our findings are in line with reported increased protein carbonylation in aged lettuce seeds [44] and *Vigna unguiculata* seeds [62]. Furthermore, amino acids like arginine, lysine, proline, and threonine—containing residues with nucleophilic centers—can react with reactive carbonyl species (RCS) derived from the 4-hydroxy-2,3-nonenal (HNE) and malondialdehyde (MDA). These reactions can potentially lead to protein damage or modifications, as observed in CS1 and CS2 seeds [63,64,65]. The accumulation of such carbonylated proteins disrupts essential metabolic pathways, including the tricarboxylic acid (TCA) cycle, electron transport chain (ETC), and glycolysis—key processes for energy production in seeds [66,67,68].

Compared to the controls, similar content in the glutelins fraction was observed in samples 4 and 10, both belonging to CS1. Additionally, in the albumins fraction, comparable content was detected in samples 5 and 8, which are part of CS2. Our findings align with previous research on *Eriochloa villosa* (Thunb.) Kunth, indicating that soluble protein content remains relatively unchanged across different storage periods. This stability is likely because the seeds predominantly rely on soluble sugars, which serve as the primary respiratory substrates and are progressively consumed during storage [69,70]. Moreover, in aged maize seeds, NADP-dependent malic enzyme (NADP-ME) catalyses the oxidative decarboxylation of malate to pyruvate, which protects against protein oxidation—especially carbonylation—during seed deterioration by providing NADPH for the antioxidant ascorbate-glutathione pathway that neutralizes ROS and other detrimental molecules accumulating as seeds age. In that way, the enzyme’s activity could contribute to better preserved total protein and individual protein fractions content in some CS1 and CS2 seed samples during natural ageing, as was reported in a study on aged *Arabidopsis thaliana* seeds [71]. However, the observed increase in total protein content and individual protein fractions in CS1 and CS2 seeds during natural ageing is generally not due to de novo synthesis. Instead, it results from a combination of factors that elevate the relative protein levels. For instance, ageing can induce cross-linking of seed proteins, leading to more stable and less degradable protein complexes. This stabilization may cause an apparent increase in measurable total protein content. In fact, the secondary structure of proteins in dry seeds across various species appears to be highly stable and remains preserved even after several decades of open storage. For example, no protein aggregation or denaturation was observed in wheat (*Triticum* spp.) seeds after 28 years of dry storage, despite a loss of viability [72]. In seeds, adenosine triphosphate (ATP) availability plays a crucial role in maintaining protein stability and supporting the activity of chaperones responsible for preserving protein integrity. As seeds age and cellular energy levels decline—primarily due to disrupted metabolism of fatty acids and glucose—ATP shortages can impair chaperone functions. This impairment reduces the seed’s ability to refold or repair damaged proteins, leading to the accumulation of misfolded or damaged soluble proteins [73].

#### 3.2.2. Analysis of Reducing and Non-Reducing Sugars

Sugar content variations in the evaluated maize seed lots may be assumed to occur without respiratory metabolism because maize seeds with less than 20% water content (dry weight basis) do not show measurable oxygen uptake [74].

The analysis of the reducing and non-reducing sugars in the evaluated seed samples reveals intricate patterns that reflect the underlying biochemical and metabolic alterations associated with seed ageing and developmental stress. Both the long-term stored CS1 samples and the CS2 samples exposed to water deficiency during the post-zygotic phase exhibited a significant average decrease in reducing sugars compared to control samples—on average, an approximate 19% glucose and ~26% maltose reduction, respectively. This reduction aligns with findings that prolonged seed storage and water deficit at the VT–R2 developmental stages significantly decrease soluble sugars, particularly glucose and maltose. This decline is attributed to enzyme inactivation and ROS-induced oxidative degradation [75]. Additionally, studies on *Vigna radiata* Wilczek have demonstrated that ROS not only directly degrade sugars and lipids but also promote lipid peroxidation, creating conditions favourable for Amadori and Maillard reactions that consume sugars and accelerate seed deterioration and loss of viability [76]. At the metabolic level, the reduction in soluble sugars indicates a decreased flux through key energy-generating pathways—glycolysis and the pentose phosphate pathway (PPP)—which impairs cellular redox balance and biosynthesis necessary for seed viability [77,78].

Interestingly, some samples shifted from this trend—CS1 sample 10 exhibited increased glucose, while both CS1 sample 10 and CS2 sample 11 expressed unchanged maltose content relative to non-aged control samples, respectively. Such fluctuations may result from residual enzymatic activity or partial hydrolysis of stored polysaccharides during ageing, leading to transient accumulation of these sugars [79].

The most pronounced changes were observed in fructose content, which increased significantly relative to controls. Long-term stored CS1 samples exhibited an average fructose increase of about 68%, with some exceeding 100%. Also, CS2 samples 5 and 7 exhibited 25.5% and 35.7% fructose content increase, respectively. High levels of free sugars (i.e., glucose and particularly fructose) contribute to damaging processes like glycation and oxidative stress [80]. They do not prevent seed ageing but rather promote it, as high levels of free sugars lead to the accumulation of Advanced Glycation End Products (AGEs), which are markers of accelerated ageing [81]. For instance, it has been demonstrated that drought contributes to the accumulation of AGEs in *Arabidopsis thaliana* leaves [82], where the majority of the 62 drought-specific glycation sites were represented by glyoxal-derived modifications, reflecting a higher increase in glyoxal (GO) equilibrium concentrations under stress conditions than other α-dicarbonyls. This up-regulation of dicarbonyl generation can be explained by drought-induced biosynthesis of osmoprotective sugar and sugar-related metabolites (so-called metabolic adjustment), as well as an increased oxidative stress [83].

Sucrose, a non-reducing sugar, serves as a substrate for the formation of storage reserves and a main nutrient and energy source for plant development [84]. It also plays an important part in carbohydrate metabolism by facilitating the stabilization of lipids and proteins in cell membranes or by promoting the vitrification of water and then the protection of cytosolic structures [9]. Aside from its reducing hexose constituents, glucose and fructose, sucrose is one of three key neutral small soluble sugars known to play a role in plant sugar signalling [85,86]. In our study, the analysis revealed an opposite trend in sucrose content between CS1 and CS2 samples compared to control samples—on average, a 6% decrease in CS1 samples and a ~13% increase in CS2 samples. A similar pattern of sucrose decrease found in long-term stored CS1 seeds was reported in aged maize [74] and Chinese cabbage seeds [87], implying that the TCA cycle was suppressed during seed ageing, blocking ATP and NADPH synthesis. Seed ageing entails increased energy expenditure, which depletes available reserves, resulting in a drop in sucrose content as the seed attempts to sustain essential metabolic functions and repair damage [88]. A notable exception was CS1 sample 1, which exhibited a 42% increase in sucrose content. This could be explained by impaired sucrose hydrolysis into glucose and fructose, likely resulting from decreased activity of soluble invertases—including cytoplasmic neutral/alkaline invertase (NI) and acid invertase (AI)—during ageing. The resulting sucrose accumulation suggests disruptions in carbohydrate hydrolysis and utilization pathways. These disruptions influence metabolic fluxes into glycolysis and the pentose phosphate pathway (PPP), since sucrose cleavage by invertase (a sucrose-degrading enzyme that produces fructose and glucose) and sucrose synthase (a sucrose-synthesizing enzyme from fructose and glucose) provide substrates for these pathways [89].

Conversely, CS2 seed samples obtained under extreme long-term water deficit exhibited a uniform increase in sucrose content compared to control samples. During seed filling, abiotic stresses hinder the import of nutrients from leaves and disrupt carbohydrate, protein, and lipid synthesis, ultimately impacting seed quality and weight [90,91]. Similar patterns of sucrose accumulation under drought stress have been observed in soybean [92] and lupin seeds [93], demonstrating that the activities of sucrose synthase and soluble invertase decrease in drought-stressed seeds [94].

#### 3.2.3. Analysis of Soluble-Free and Insoluble-Bound Phenolics

All long-term stored CS1 samples displayed a noticeable lightening of seed colour compared to the dark orange hue of CS2 samples and non-aged controls, initiating detailed physiological and biochemical analysis that may explain this phenomenon. Seeds can either darken or lighten during long-term storage, depending on the plant variety and especially the plant species, with a seed’s antioxidant profile playing a significant role. In some cases, they can develop tiny holes or air-filled spaces resulting from internal damage caused by prolonged cold storage. The fading of seed colour during ageing reflects underlying biochemical degradation, loss of pigments, and structural deterioration within the seed. These processes often coincide with reductions in seed weight, as observed in all CS1 samples, indicating compromised internal composition and physical integrity. Therefore, seed colour lightening can serve as a visible indicator of the internal deterioration that also affects weight, providing a visual cue for seed viability and vigour during storage. In maize seeds, phenolic compounds—abundant in the pericarp, aleurone layer, endosperm, and embryo [95]—play a crucial role. These compounds are potent antioxidants that help preserve seed integrity and coloration throughout storage. During seed ageing, phenolics degrade, leading to diminished antioxidant protection and subsequent biochemical and structural deterioration. This breakdown manifests visually as seed colour lightening due to pigment degradation and loss, and it is associated with reductions in seed weight, reflecting compromised internal reserves and structural components. Therefore, understanding the dynamics of phenolic compounds is essential for elucidating the biochemical mechanisms behind seed ageing, colour changes, and physical deterioration [96].

During seed aging, analysis of phenolic compounds revealed that both CS1 and CS2 samples maintained similar levels of total soluble-free phenolics (FPs) and insoluble-bound phenolics (IBPs) compared to their respective non-aged controls. Only a few samples exhibited sample-specific increases in either FPs or IBPs, which align with those reported on *Trifolium repens*, a longer-lived perennial, for which storing seeds for up to 40 years in dark, dry, and cool conditions (15–20 °C) leads to an increase in total phenolic content compared to fresh controls [40]. Both FPs and IBPs are crucial for plant physiology. FPs are often involved in immediate cellular functions, while IBPs contribute to structural stability and serve as reserves that can be released under stress or during germination [97]. The observed increases of up to 13.7% in FPs and 9.3% in IBPs suggest an active response to ageing, indicating that seed deterioration—an intrinsic stress condition—often triggers phenolic biosynthesis or release as part of the plant’s antioxidative defence system [70]. In seeds, both FPs and IBPs play roles in regulating germination, frequently exerting inhibitory effects by blocking or slowing down the process. The accumulation of soluble-free and insoluble-bound phenolics during seed ageing or under exogenous stress conditions can create an environment that inhibits key enzymes such as phosphatases—responsible for reversible protein phosphorylation, a vital signalling process regulating hormone responses, metabolic activation, and cellular homeostasis during germination—and prolyl aminopeptidases, which are involved in protein processing and turnover and provide essential nutrients for the developing seedling [98]. Since the outer layers of mature seeds are rich in phenolic compounds, their content and nature appear to serve as indicators of their involvement in germination inhibition [99].

In this study, we examined the three most common hydroxycinnamic acids: *p*-coumaric acid, ferulic acid, and caffeic acid. We detected their bound forms in all maize seed samples; however, the free form of caffeic acid was exclusively present in genotype MRIZP13695. Phenolic acids play an important role in antioxidant protection, structural integrity via cell wall strengthening, and defence mechanisms [100]. During seed ageing, the activation of the phenylpropanoid biosynthetic pathway leads to increased levels of phenolic acids. These compounds are not only strong antioxidants but also act as signalling molecules that trigger and regulate protective responses, ultimately helping to prolong seed viability and delay deterioration [101]. Compared to the non-aged control samples, CS2 samples exhibited uniform increases in both soluble-free and insoluble-bound FA content. There was a predominant increase in insoluble-bound *p*-CouA, while soluble-free *p*-CouA showed a highly variable increase of approximately 23%—observed in samples 2 and 5. Additionally, soluble-free CA increased by about 31%, and insoluble-bound CA by ~17%, with both increases observed in sample 5.

However, long-term stored CS1 samples and CS2 sample 11 revealed a significant surge in free FA, with increases reaching up to 367% and 106%, respectively. This overaccumulation of soluble-free and insoluble-bound phenolic acids can negatively impact seed viability and vigour. Elevated levels of FA, *p*-CouA, and CA may interfere with germination by binding to enzyme active sites or chelating essential metal cofactors, thereby inhibiting crucial enzymes involved in seed germination [98]. While phenolics are generally recognized for their antioxidant properties, in high concentrations they can become prooxidants, contributing to oxidative stress. During seed ageing, phenolic acids may undergo auto-oxidation, generating ROS such as hydrogen peroxide and superoxide radicals [102]. Elevated ROS levels can damage lipids, proteins, and DNA within seed tissues, impairing cellular integrity and ultimately reducing seed viability [103]. Soluble-free FA can increase cell wall rigidity by participating in oxidative cross-linking with cell wall polysaccharides, forming feruloyl-polysaccharide complexes and influencing lignin interactions [104]. Although this reinforcement might offer some protective benefits, excessive cross-linking diminishes cell wall plasticity. This rigidity hampers the seed’s ability to undergo necessary morphological changes during germination, such as radicle protrusion. As a result, seedling emergence is impaired, negatively affecting overall germination success [105].

During seed ageing, a decreased accumulation of soluble-free and insoluble-bound phenolic acids can also have negative consequences, primarily compromising the seed’s defence mechanisms and structural integrity. Phenolic acids serve as natural antioxidants within seeds, effectively scavenging ROS generated during seed maturation, dormancy, and germination [106]. For instance, studies on millet have shown that insoluble-bound FA and *p*-CouA exhibit remarkable ROS scavenging activities, ranging from 38% to 99% [107,108]. In our research, however, most long-term stored CS1 seed samples demonstrated an approximately 18% reduction in *p*-CouA content compared to non-aged controls. This decline suggests a weakened antioxidant defence against the effects of ageing. Reduced phenolic acid levels diminish the seed’s ability to neutralize ROS, thereby increasing vulnerability to oxidative stress [109]. Furthermore, when ROS levels increase—especially under hydration conditions much lower than those typically found in stored seeds—lipoxygenases (LOXs) can catalyse lipid peroxidation, leading to cell membrane damage. Such damage results in the loss of membrane integrity, leakage of cellular contents, and impaired metabolic functions, all of which negatively impact seed germination and seedling development [110]. Our findings suggest that the capacity of CS1 seeds to inhibit LOX activity—a key factor in lipid peroxidation and seed aging—may be significantly compromised due to decreased *p*-CouA levels [111]. Additionally, a reduction in phenolic content can impair signal transduction and stress response pathways. This impairment hampers the activation of ROS-sensitive signalling mechanisms and stress-responsive transcription factors such as WRKY and NAC, leading to diminished expression of protective and repair genes. Moreover, decreased phenolic acids can disrupt hormonal signalling pathways involving abscisic acid, salicylic acid, and jasmonic acid, further weakening the seed’s ability to respond effectively to oxidative and environmental stresses [112,113].

#### 3.2.4. Analysis of Tocopherols and Carotenoids

Tocopherols, a class of vitamin E compounds, serve as crucial antioxidants in seeds, safeguarding polyunsaturated fatty acids (PUFAs) from oxidative damage during storage and ageing [114]. The specific composition and balance of different tocopherol isomers significantly influence a seed’s resilience to ageing. Generally, higher levels of *α*-tocopherol are associated with enhanced seed longevity, while *γ*-, *β*-, and *δ*-tocopherols provide additional antioxidant support, particularly under oxidative stress conditions [115]. In our study, we observed that prolonged seed storage—up to 37 years—led to a consistent net decline in tocopherol content: approximately 28% for *α*-T, 25% for *β*+*γ*-T, and a striking 52% for *δ*-T in CS1 samples. This decline suggests that ageing during extended storage may cause down-regulation of the vte1 and vte2 genes, which encode key enzymes in tocopherol biosynthesis—tocopherol cyclase (TC) and homogentisate phytyltransferase (HPT), respectively. Suppressed gene expression often results from oxidative stress during ageing, damage to the transcriptional machinery, and epigenetic modifications—DNA methylation and histone modifications. The HPT, encoded by the gene vte2, catalyses the condensation of homogentisic acid (HGA) and phytyl diphosphate (PDP), precursor compounds for tocopherol biosynthesis in seeds, to form 2-methyl-6-phytyl-1,4-benzoquinol (MPBQ), the first committed step in tocopherol (vitamin E) biosynthesis. The subsequent conversion of MPBQ into tocopherols (like *γ*-T and ultimately *α*-T) involves further modifications, including methylation and cyclization, with the latter step mediated by TC, encoded by the gene vte1 [116,117]. Accordingly, our findings are supported by those from studies on *Arabidopsis thaliana*, underscoring the importance of these genes: plants lacking vte2 were completely deficient in all tocopherol derivatives and their precursors, highlighting vte2’s essential role in tocopherol biosynthesis [118]. Additionally, studies have shown that transgenic approaches employing antisense RNA to suppress vte2 mRNA levels resulted in a tenfold decrease in tocopherol content in *A. thaliana* seeds. Similarly, vte1 mutants, which lack a functional TC enzyme, cannot produce tocopherols and instead accumulate the intermediate DMPBQ—the uncyclized precursor to γ-tocopherol [119].

On the other hand, the analysis revealed that the majority of CS2 samples experiencing severe water deficit during the post-zygotic phase showed a significant increase in *α*-T content, averaging 22.5%. Additionally, CS2 samples 5 and 8 exhibited elevated levels of *γ*- and *δ*-T—by 16% and up to 25% compared to non-aged seed samples. Water deficit induces cellular dehydration and disrupts normal metabolic functions, leading to the overproduction of ROS and reactive nitrogen species (RNS). These reactive molecules trigger the seed’s antioxidant defences, including the biosynthesis of tocopherols, as a protective mechanism [120]. Studies on abscisic acid (ABA)-deficient *Arabidopsis thaliana* mutants have shown that tocopherol synthesis during water deficit stress, particularly in seeds, is predominantly regulated via ABA-independent signalling pathways [121]. The increased accumulation of tocopherols—especially *α*- and *γ*-T—in CS2 samples can be attributed to the upregulated activity of TC and HPT, the key enzymes involved in tocopherol biosynthesis, contributing to limited enzymatic and non-enzymatic lipid oxidation under oxidative stress imposed by water deficit [122]. Conversely, a decrease in tocopherol content in some CS2 samples may indicate that, under water deficit stress, metabolic pathways prioritize channelling homogenate—a precursor or building block in the synthesis of tocopherols—into the Krebs cycle primarily for energy production rather than tocopherol synthesis. This shift likely results in lower tocopherol accumulation, reflecting a strategic allocation of resources to maintain cellular energy needs during stress [123].

In our study, analysis of carotenoid content revealed that prolonged cold storage—up to 37 years—led to a significant average reduction in *β*-carotene and lutein+zeaxanthin contents by approximately 89% and 75%, respectively, compared to non-aged control seeds. Because carotenoids contain conjugated double bonds, they are highly susceptible to oxidative degradation, which is a primary factor behind the decline in carotenoid content during seed aging [124,125]. This oxidative process is often triggered by increased lipid peroxidation and the activity of lipoxygenases (LOXs) [126,127]. It is plausible that during seed ageing, elevated ROS levels generated by LOXs activity may cause direct oxidative modifications of phytoene synthase (PSY), a major rate-limiting enzyme in the carotenoid biosynthesis pathway. The activity of PSY largely determines the metabolic flux toward carotenoid biosynthesis. Oxidative stress and metabolic deterioration in CS1-aged seeds could impair PSY’s catalytic function and stability, potentially leading to increased proteolytic degradation that lowers enzyme levels. Additionally, seed ageing might influence the transcriptional regulation of PSY genes, down-regulating their expression [128]. The decline in PSY activity due to ageing consequently impairs carotenoid biosynthesis, which negatively affects the vigour and quality of CS1 seeds. It has been reported that ABA is a key factor involved in PSY down-regulation during seed ageing. Elevated ABA levels may repress PSY gene transcription and activate stress-responsive pathways that suppress carotenoid biosynthesis [129]. This finding was confirmed by physiological and proteomic analyses of artificially aged *Brassica napus* seeds, which showed increased ABA content and highlighted its role in initiating seed ageing [130]. Furthermore, we cannot exclude the possibility that increased expression and activity of carotenoid-degrading enzymes—carotenoid cleavage dioxygenases (CCDs) and 9-cis carotenoid cleavage dioxygenases (NCEDs)—may contribute significantly to the net reduction in carotenoids in CS1 maize seeds during prolonged storage. This aligns with findings from linkage mapping and genome-wide association studies (GWAS), which identified CCD4 as a major locus negatively regulating carotenoid levels in *Arabidopsis* seeds [131].

In our study, analysis of carotenoid content also revealed that CS2 samples subjected to severe water deficit stress during the post-zygotic phase exhibited a similar, although less pronounced, decrease in *β*-carotene and lutein+zeaxanthin by ~38%, i.e., ~40%, respectively, compared to non-aged control seeds. This reduction suggests that water deficit conditions influence carotenoid biosynthesis. Research on the PSY gene family, which is conserved in the *Poaceae*, including maize, indicates that under water deficit stress—similar to that experienced by maternal plants of CS2 seeds—increased expression of the PSY3 gene leads to elevated levels of carotenoid intermediates. These intermediates subsequently enhance the expression of the nonheme diiron *β*-carotene hydroxylase (HYD) and NCED genes, both of which are involved in the biosynthesis of ABA, a hormone crucial for plant stress responses. Therefore, the observed decrease in carotenoid content under drought conditions may reflect a shift in maternal plant metabolism regarding the carotenoid biosynthesis pathway, resulting in decreased carotenoid content but increased ABA production, which is vital for surviving under stress conditions [132].

Additionally, it can be hypothesized that water deficit stress reduces photosynthesis in the maternal plant, limiting the supply of precursors and signalling molecules necessary for carotenoid biosynthesis in developing CS2 seeds—non-photosynthetic tissues that depend on the parent plant for these compounds. Water deficit stress decreases CO_2_ uptake, thereby slowing photosynthesis and reducing the production of isoprenoid precursors—isopentenyl pyrophosphate (IPP) and its allylic isomer dimethylallyl pyrophosphate (DMAPP), which are essential building blocks for carotenoids [133]. Consequently, this reduction in precursor supply may directly hinder carotenoid synthesis in the CS2 seeds.

#### 3.2.5. Analysis of Total Antioxidant Capacity

Antioxidants exert their effects through various mechanisms, including scavenging free radicals, sequestering transition metal ions, decomposing hydrogen peroxide or hydroperoxides, quenching active prooxidants, and repairing biological damage [134,135]. Accordingly, we carefully selected the antioxidant assay method [136] to assess whether the evaluated antioxidants are effectively rendered soluble and accessible within seed tissues. Such accessibility could enhance antioxidant activity and improve the seed’s capacity to withstand ageing-related deterioration. Our results indicated that the duration of cold storage did not significantly influence the TAC. However, analysis of the biochemical parameters revealed limitations in measuring TAC. While TAC reflects the total antioxidant pool in aged seeds, it does not necessarily indicate the functional or protective status of antioxidants—their efficiency in neutralizing ROS/RNS—nor does it reveal the extent of molecular damage. The high TAC values observed in CS1 and CS2 seed samples, which were nearly comparable to those of non-aged control seeds, may suggest a state of elevated oxidative stress leading to increased antioxidant consumption. The cellular homeostatic mechanisms regulate the total antioxidant activity; modifying the levels of one antioxidant often triggers compensatory changes in others, maintaining the overall capacity [137]. Recent research on seed longevity and ageing [39] underscores the significant role of oxidative stress in seed ageing, as demonstrated by genome-wide and reverse genetics studies on *Arabidopsis thaliana*. These studies identified genes involved in ROS metabolism and detoxification as key factors influencing seed longevity [138]. Reactive oxygen species, generated from the partial reduction of oxygen, can cause oxidative damage when their levels exceed the homeostatic capacity of the seed antioxidant system, particularly under stress conditions—seed ageing and water deficit [139].

### 3.3. Correlations

#### 3.3.1. The Total Protein, Protein Fractions and Seedling Growth Parameters

The observed correlations across the seed groups underscore that, despite differences in their stress histories—prolonged cold storage (CS1 seeds) and exposure to extreme water shortage during the post-zygotic phase (CS2 seeds)—total protein and glutenins fraction are closely linked to early seedling growth parameters. These include positive associations with root and shoot lengths, as well as the SVI-I, which combines germination percentage and total seedling length—particularly in CS2 seeds. In non-aged control seeds, higher total protein and glutelins contents are strongly correlated with seedling vigour, reflecting their vital roles in supporting germination and early development. During the imbibition phase of seed germination, storage proteins are broken down to provide nutrients and energy required, inter alia, for the translation of stored mRNA and the biosynthesis of new proteins with specific functions [140]. Initially, this protein degradation leads to a decrease in seed dry weight, which is supported by highly significant negative correlations between total protein, i.e., the glutelins fraction content, and the seedling vigour index that incorporates total seedling dry weight (SVI-II). Ultimately, the net change in dry weight results from a balance between the breakdown of stored nutrients and the synthesis of new structural and functional proteins required for seedling development [141]. All these align with previous findings indicating that SSPs provide essential amino acids and nutrients necessary for seedling growth [57,142]. However, in 37-year-old CS1 seeds and five-year-old CS2 seeds, these relationships weaken or invert due to oxidative modifications like ROS-induced carbonylation, which impair glutelins function despite stable or increased total protein content [60,143]. Such modifications, including the cross-linking of storage proteins, can lead to the formation of more stable yet less functional protein complexes, thereby disrupting key metabolic pathways like glycolysis and the TCA cycle, which are essential for vigour [144]. In CS1 and CS2 seeds, the negative correlations observed between total protein or glutelinss contents and SVI-II further suggest that accumulated protein damage or modified proteins hinder growth, aligning with evidence that oxidative damage and protein carbonylation reduce seed vigour rather than total protein reserves per se [145]. Accordingly, total protein content in aged seeds may appear stable or even elevated, but their functional integrity is compromised. This leads to reduced seed vigour, supporting the evidence that protein quality and post-translational modifications, rather than quantity alone, govern seed ageing and early seedling performance [146].

#### 3.3.2. The Reducing and Non-Reducing Sugars and Seedling Growth Parameters

The correlations observed across the three seed groups suggest that natural ageing—whether resulting from prolonged cold storage or water deficit stress during seed development—significantly affects sugar metabolism and its relationship with seed vigour and seedling growth. In control seed samples, higher fructose contents positively correlated with seedling length and the SVI-I index, indicating an intact sugar metabolism that supports seedling development. These findings align with genetic studies on *Arabidopsis thaliana*, which have revealed that fructose is involved in multiple regulatory layers governing early seedling establishment after seed germination, with its signalling acting downstream of the ABA pathway [147]. Conversely, these positive correlations diminish or even invert in CS1 and CS2 seeds, suggesting that accumulated fructose may contribute to oxidative damage via glycation and ROS-mediated degradation, ultimately impairing seedling growth [80,81].

Fructose serves as a readily available substrate for cellular respiration during germination, being metabolized via glycolysis to generate ATP and support early seedling development [148]. The positive correlation between fructose content and SVI-II observed in five-year-old CS2 seeds indicates that higher free fructose levels are associated with enhanced early growth potential. Conversely, control seeds showed a significant negative correlation, suggesting an over-reliance on free fructose alone. This reliance might lead to rapid depletion of energy reserves, potentially limiting sustained seedling growth. Despite a high germination percentage, control seeds may exhibit lower SVI-II values because the initial energy supplied by free fructose does not fully compensate for the depletion of stored reserves necessary for continued seedling development and dry matter accumulation [149].

When examining sucrose content, opposite correlation patterns emerged compared to fructose. In control seeds, significant negative correlations were observed between sucrose levels and seedling length, i.e., SVI-I. Healthy, non-aged seeds with an inherently lower sucrose content (in general, ~1–3% of total dry weight in mature maize seeds not bred for sucrose content) tend to produce vigorous seedlings with faster elongation due to intact metabolic pathways for energy conversion; a high sucrose content may negatively affect germination and seedling development by acting as a signalling molecule that may cause developmental arrest [150,151]. The seeds rely on stored lipids, which are efficiently broken down into sugars for energy during germination—a process that appears less efficient in aged seeds with impaired metabolism [88]. Interestingly, the only significant positive correlation exhibited by control seeds was observed between sucrose content and SVI-II. Sucrose acts as a source of sugars for metabolic transformations, respiration, and osmotic regulation during early development [152]; however, the inherently lower sucrose content in the evaluated maize genotypes may suggest a reliance on free fructose, which alone does not fully compensate for the depletion of stored reserves necessary for continued seedling growth and dry matter accumulation [149].

CS1 and CS2 seeds exhibited weaker or non-significant relationships between sucrose content and seed vigour, i.e., seedling growth. Sucrose generally promotes overall growth—including cell elongation and division—through complex hormonal and signalling pathways [153]. The contrasting trends in sucrose content—decreasing in CS1 and increasing in CS2—reflect disruptions in carbohydrate hydrolysis pathways, likely influenced by their stress history and enzyme activity levels [88]. The only significant trend in observed relations was expressed by aged CS1 seeds, revealing that decreased sucrose content resulted in reduced seedling shoot length [154].

#### 3.3.3. The Soluble-Free, and Insoluble-Bound Phenolics and Seedling Growth Parameters

The contrasting relationships observed between phenolic compounds and seedling growth performance can be explained by physiological and biochemical mechanisms, considering the functional roles of IBPs and FPs in seed physiology and early seedling development [155]. In control and CS2 seeds—both exhibiting high germination percentages—high IBPs content has been associated with strong antioxidant capacity. This helps protect embryonic tissues from oxidative stress during germination, thereby supporting early seedling growth (i.e., root and shoot elongation) and contributing to a higher SVI-I [106]. However, aged CS1 seed samples, which expressed compromised physiological quality, show that increased insoluble-bound FA content correlates with more pronounced restrictions in seedling length and SVI-I reduction. This is because FA contributes to cell wall stiffening via oxidative cross-linking, physically impeding cell expansion and growth [156,157]. Additionally, FA inhibits key enzymes necessary for germination and growth—hydrolytic enzymes—that are required for nutritional reserves mobilization. This inhibition results in a decreased energy supply for the developing seedling, consequently stunting root and shoot growth, with root elongation often being more affected [158,159]. Moreover, decreased soluble-bound *p*-CouA and CA content indicated that most CS1 seed samples have compromised antioxidant defences, making them more susceptible to oxidative damage, lipid peroxidation, and membrane degradation—all of which eventually hinder the seedlings’ development [109]. During germination, seeds undergo intense cellular respiration to generate the ATP necessary for enzymes that break bonds between IBPs and other cell wall components like polysaccharides. This process releases IBPs and facilitates wall loosening, both of which are essential for cell expansion and division during seedling growth. Seeds with high IBP content (control and CS2 seeds) require higher metabolic activity to overcome the inhibitory effects of phenolic compounds—reflected in higher respiration rates—leading to increased consumption of stored reserves. This heightened metabolic demand depletes the seed’s dry reserves, resulting in reduced seedling vigour and biomass accumulation, as evidenced by significant negative correlations between IBPs content and SVI-II [160].

In contrast, in control and CS2 seeds, higher FPs and free-soluble *p*-CouA contents correlated negatively with seedling root and shoot length, as well as SVI-I. Phenolic compounds have been shown to have both a low-dose stimulatory and a high-dose inhibitory effect [161]. During the germination phases, IBPs that are covalently bound to cell wall components like cellulose, pectin, lignin, hemicellulose, proteins, or carbohydrates [162] are converted into FPs by endosperm degradation [163]. However, in non-aged control seeds, the soluble-free *p*-CouA content is likely below the inhibitory threshold; therefore, it has no negative impact on seedling growth or SVI-I. It is possible that the negative correlation simply reflects the fact that in seeds with a higher phenolic acid content—which indicates a higher stress response—germination may still be in progress or have somewhat different growth rates, but growth is not inhibited. In comparison to the controls, CS2 seeds that experienced a severe water deficit during the post-zygotic phase showed genotype-dependent variation in soluble-free *p*-CouA content [164]. They showed no reduction in seedling growth or SVI-I performance, but a significant decrease in SVI-II performance. In the case of CS2 seeds, the negative correlation between FPs, i.e., soluble-free *p*-CouA, and early seedling growth, i.e., SVI-I [165], and the positive correlation between soluble-free *p*-CouA and SVI-II [166] may therefore be related to “single-generation stress single-generation stress memory”. This implies that the seed’s epigenome has been modified by the prior water deficit stress experienced by the maternal plant to produce these protective compounds, even if it does not directly affect seedling growth. Our conclusion is supported by the general principle that plant stress responses are influenced by epigenetic regulation, specifically histone modification (methylation and acetylation) [167]. This has been documented in a number of plant species, including maize [168], rice seedlings [169], and *Arabidopsis* [170], demonstrating how DNA methylation or histone modifications can increase on stress-inducible genes. Conversely, CS1 seeds showed a significant positive correlation between FPs and both the SVI-I and SVI-II, which is unquestionably the result of damage—an oxidative response—not a beneficial relationship. The marked decline in seedling vigour indices results directly from the combined effects of intensive oxidative damage, membrane leakage, and enzymatic dysfunction caused by aging. The unchanged FPs content in CS1 seeds, compared to controls, cannot compensate for these widespread damages. These findings align with previous research on the relationship between the content of total phenolics in exudates and the germination capacity of maize seed during accelerated ageing [171].

#### 3.3.4. The Tocopherols and Carotenoids and Seedling Growth Parameters

Vitamin E is the most efficient non-enzymatic inhibitor of lipid oxidation in cell membranes, which is one of the main reasons for seed ageing. This vitamin comprises a family of eight naturally occurring tocochromanols, including *α*-, *β+ϒ*-, and *δ*-tocopherols, as well as four corresponding tocotrienols [172]. Vitamin E acts as a chain-breaking antioxidant during free radical-mediated lipid oxidation, owing to its ability to react with free radicals within cell membranes and other lipid-rich environments [173]. Consequently, the loss of tocopherol protection has been associated with seed deterioration [122]. This aligns with our findings, which show that prolonged storage induces age-related oxidative stress, leading to a significant decline in *α-* and *β+ϒ*-T contents in CS1 seeds. This decline compromises the seed’s antioxidant defences and renders membranes more susceptible to oxidative damage during germination. Although the correlations observed were not statistically significant, the reduction in *α-* and *β+ϒ*-T content clearly impaired seed germination and seedling growth, as reflected in seedling vigour indices, compared to control seeds. In response to water deficit during the post-zygotic phase, CS2 seeds exhibited upregulation of tocopherol biosynthesis, resulting in higher *α*-T content relative to control seeds. This response is likely mediated by stress signalling pathways that activate the expression or activity of key enzymes involved in tocopherol biosynthesis, thereby enhancing the seed’s antioxidant capacity to counteract lipid oxidation and maintain membrane integrity [120,121]. The increased *α*-T content in CS2 seeds suggests an adaptive defence mechanism against oxidative stress, aimed at preserving seed and seedling viability. This is supported by highly significant positive correlations with seedling elongation and the SVI-I. Our findings align with those from studies on *Pinus sylvestris* seeds, which indicate that *α*-T is the primary component of the vitamin E family involved in protecting aging seeds from oxidative stress [174]. However, seedling root and shoot length—and consequently SVI-I—showed a negative correlation with *β+ϒ*-T content in CS2 seeds. This observation aligns with reports on early seedling development in barley [175]. The contrasting trends observed in correlations involving *α*- and *γ*-T reflect findings from studies on transgenic Arabidopsis, soybean, and maize seeds overexpressing *γ*-T methyltransferase (*γ*-TMT). These transgenic seeds exhibit a tocopherol profile dominated by α-T instead of γ-T. Notably, germinating transgenic soybean seedlings—but not dormant seeds—have been shown to contain less lipid oxidation products than their wild-type counterparts, suggesting that *α*-T provides better protection against lipid oxidation during photomorphogenesis [176,177,178].

The relationship between *β*-carotene content and seedling growth is not always linear. The naturally occurring high *β*-carotene content found in control seeds (averaging approximately 15 μg g^−1^ DW) may not be optimal for maximizing early seedling elongation. This is evidenced by the strong, statistically significant negative correlation between *β*-carotene content and seedling growth parameters and the resulting SVI-I. Accordingly, it can be hypothesized that the conversion of *β*-carotene into retinaldehyde involves carotenoid cleavage dioxygenases (CCDs)—enzymes that cleave carotenoids to produce apocarotenoids. These molecules are known to influence various physiological processes, including seed germination, seedling elongation, and hormone signalling pathways [179,180]. Conversely, long-term seed storage accelerates the oxidative degradation of *β*-carotene, as its conjugated double bonds are highly susceptible to oxidative cleavage, resulting in a significant reduction in its content [124,125]. The observed negative correlations suggest that as carotenoid content declines due to oxidative breakdown, early seedling growth—particularly shoot development—becomes substantially impaired. The significant negative correlations between the same parameters were found in CS2 seeds, which, despite showing a significant decrease in *β*-carotene content, still germinated successfully and produced seedlings with preserved root and shoot elongation, as well as high SVI-I. This indicates the presence of compensatory mechanisms, such as efficient resource reallocation that prioritizes vital processes for germination. These include directing stored nutrients toward embryo growth, cell division, and enzyme activation necessary for germination [181,182,183,184]. Additionally, key metabolic pathways involved in early growth—such as glycolysis and the TCA cycle for energy production [185], amino acid biosynthesis pathways like the shikimate and glutamine pathways [186,187], and cell wall biosynthesis pathways responsible for synthesizing cellulose and hemicellulose [188]—are likely activated to support seedling development. The relationship between *β*-carotene content and seed vigour, germination capacity, and SVI-II persists across different environmental conditions, resulting in a positive correlation between these traits in both seed groups (control and CS2 seeds with water-stress history). This means that even under stress, the relative differences in *β*-carotene content among seeds could still predict their success in germination and seedling development [189,190].

## 4. Materials and Methods

### 4.1. Plant Material

In compliance with the Genebank Standards for Plant Genetic Resources for Food and Agriculture [191], the MRIZP gene bank ex situ maize collection is kept in medium-term conditions at +4 °C, an approximate relative humidity of 40%, and seed moistness of 11%.

Four introduced advanced maize cultivars were evaluated in this study, and each of these provided three seed lots for analysis: the original seed lot harvested in 1985 (i.e., 37 years of cold storage; CS1), the original seed lot regenerated in 2017 (i.e., five years of cold storage; CS2), and the original seed lot freshly harvested in 2022 (i.e., control—a proxy of how freshly harvested, non-aged seeds should perform). The information on the evaluated maize accessions is provided in Table 7.

**Figure 4:**
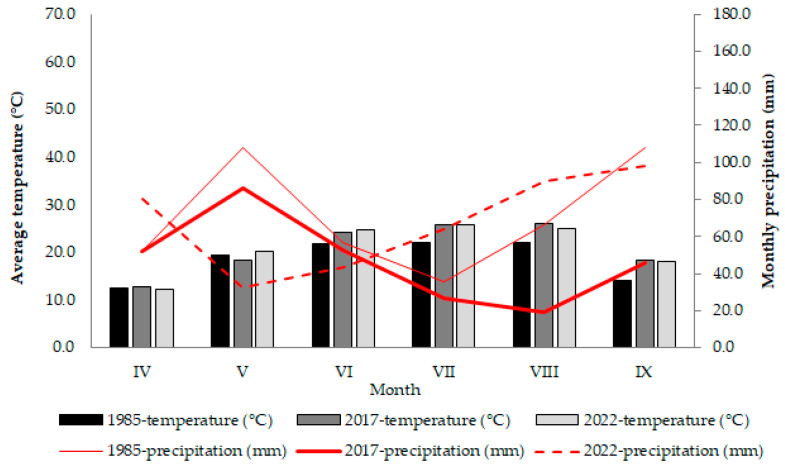
The Walter-Lieth chart of the climatic variables at Zemun Polje for the given years of regeneration (i.e., 1985, 2017, and 2022).

The genotypes (twelve samples in total) were regenerated at the location of Zemun Polje, Serbia (longitude 20°18′60.00″ E, latitude 44°51′59.99″ N, altitude 80 m), characterised by a Calcaric Chernozem with a silty loam texture. The genotypes were multiplied manually via pair crossing (i.e., full sibling). The method of technical isolation was exploited to avoid gene flow or contamination. Routine, standard cropping practices were applied in the autumn and in the spring before sowing. Also, weeds, pests, and diseases were adequately controlled. In addition, the environmental conditions during the vegetative period (April–September) are given in Figure 4. Based on both temperature and monthly precipitation, it can be observed that the agro-ecological conditions during the vegetative period (April–September) were optimal for maize growth in 1985 and 2022. Additionally, the average temperature during the vegetative period in 2017 was also favourable at 21 °C. However, there was an extremely low amount of precipitation during two critical phenophases: the flowering phase, a twenty-day period in June–July with only 17.4 L m^−2^ of rainfall, and the grain filling phase, covering the entire July–August period with 45.9 L m^−2^ of rainfall. Considering that the estimated precipitation needed for optimal maize growth in the region where seed regeneration was conducted is approximately 459.0 L m^−2^, the water deficit stress experienced in 2017 can be considered as both long-term and severe. Ears of the maize gene bank accessions were manually harvested at full physiological maturity (R6 phenophase).

### 4.2. Assays for Seed Germination and Seedling Vigour

The International Seed Testing Association (ISTA) Rules [192] were followed while performing the standard germination test. The between-papers approach was conducted at 20/30 °C and an 8/16 h (light/dark) photoperiod. A germination assay was applied to 50 seeds per accession, in three biological replicates. The seedlings were evaluated following four days of germination (i.e., the first count) and seven days of germination (i.e., the standard germination), respectively.

After completing the standard germination test, seedling growth parameters were assessed. From each of the three replicates, ten normal seedlings at the stage of the first foliage leaf emerging from the coleoptile—each with a root system consisting of a primary root and secondary roots—were randomly selected for evaluation, and the results were averaged. For each seedling, the length (L) and fresh weight (FW) of the shoot, root, and seed residue were measured separately. Subsequently, the same seedlings were oven-dried at 40 °C for 12 h until reaching a constant weight, and their dry weights (DW) were recorded.

Seedling Vigour Index I (SVI-I) and Seedling Vigour Index II (SVI-II) are considered the relevant indices of seed performance potential both in the field and in storage and are calculated [193] as follows:

(1) SVI-I=Germination (%)×Average seedling length (cm)

Seedling length refers to the total length of root and shoot per plant, respectively.

(2) SVI-II=Germination (%)×Average seedling dry weight (mg)

Seedling dry weight refers to the total dry weight of root, shoot, and seed residue per plant, respectively.

### 4.3. Biochemical Markers

#### 4.3.1. Sample Preparation and Chemicals Used for the Analyses

The grain of the evaluated maize samples was milled (Perten MILL 120 CE, Perten Instruments in Hägersten, Sweden) into flour of particle size < 500 μm and kept at −20 °C until the analyses. All chemicals and solvents used were of analytical or High-Performance Liquid Chromatography (HPLC) grade and were purchased from Sigma-Aldrich (Steinheim, Germany). Ultrapure water (Thermo Fisher TKA MicroPure water purification system), with a conductivity of 0.055 µS/cm, was used throughout the experiment.

#### 4.3.2. Analysis of Total Protein

The total protein content was determined according to the Micro-Kjeldahl procedure [194] on the AutoKjeldahl Distillation Unit K-350 and Speed Digester K-439 (BÜCHI, Labortechnik, Switzerland). The total protein content was calculated from the nitrogen content determined by the Micro-Kjeldahl method using a nitrogen-to-protein conversion factor of 6.25 and expressed as the percentage of protein in dry matter (%).

#### 4.3.3. Analysis of Protein Fractions

Different protein fractions were obtained by successively extracting defatted flour with a series of solvents (1:10, *w*/*v*), following the Osborne procedure as described by Lookhart and Bean [195], albeit with some modifications. Albumins, globulins, α-zeins, and glutelins (G3-glutelin) fractions were extracted by distilled water, 0.5 M NaCl, 70% ethanol, and 0.0125 M borate buffer, pH 10, containing 5% sodium dodecyl sulphate, respectively. Each protein fraction was extracted using three rounds of repeated stirring at 4 °C for 30 min, followed by centrifugation at 20,000× *g* for 15 min (Dynamica Velocity 18R Refrigerated Benchtop Centrifuge, DKSH New Zealand Limited, Milson, New Zealand). The protein content in each fraction was calculated from the nitrogen content determined by the Micro-Kjeldahl method using a nitrogen-to-protein conversion factor of 6.25 and expressed as the percentage of dry matter (%).

#### 4.3.4. Analysis of Sugars

The extraction of sugars from 1 g of maize flour with ultrapure water was done in three stages (10, 5, and 5 mL) by vortexing for 3 min, followed by centrifugation at 6000× *g* for 5 min, respectively [196]. A total of 1 mL of water extract was mixed with Carrez I and Carrez II solutions (50 μL each), and after stirring, the mixture was centrifuged at 6000× *g* for 3 min. The clear supernatants were filtered through HyperSep Retain PEP SPE columns (Thermo Fisher Scientific, Waltham, MA, USA), preconditioned by washing with 1 mL of methanol and then 1 mL of water. Analysis of sugars was performed on a Thermo Scientific Ultimate 3000 HPLC system consisting of a quaternary pump, an autosampler, a column oven, and a refractive index detector (RID). After injecting 5 μL of extract, sugars were separated on a Thermo Scientific Hypersil GOLD Amino column (150 mm × 3 mm, 3 µm) conditioned to 30 °C [197].

An isocratic elution with a mobile phase consisting of 87% acetonitrile in water (*v*/*v*) at a flow rate of 0.7 mL/min was used, and the chromatographic run was completed in 30 min. Sugars were identified by comparing their retention times with those of corresponding standards and by spiking the samples with the appropriate standard, and quantified using the Thermo Scientific Dionex Chromeleon software package (version 7.2).

The results are expressed as the percentage of sugar in dry matter (%).

#### 4.3.5. Extraction of Soluble-Free and Insoluble-Bound Phenolic Compounds

The soluble-free and insoluble-bound phenolic compounds in the evaluated maize samples were extracted according to the procedure described by Antoine et al. [198]. For the extraction of soluble-free phenolic compounds from 0.5 g of flour, 20 mL of an acetone/methanol/water solvent system (7:7:6, *v*/*v*/*v*) was used. The insoluble-bound phenolic compounds in the solid residue were released by alkaline hydrolysis for 4 h at room temperature using 4 N NaOH before the extraction. After adjusting the pH to 2.0 with 6 N HCl, free supernatants and hydrolysates were extracted with ethyl acetate and diethyl ether (1:1, *v*/*v*) four times. The combined phenolic extracts were evaporated under the N_2_ stream at 30 °C to dryness, and the final residues were re-dissolved in 1.5 mL of methanol. After filtering through 0.45 μm nylon syringe filters (Phenomenex, Torrance, CA, USA), the prepared samples were kept at −20 °C until HPLC analyses of total phenolic compounds and phenolic acids.

#### 4.3.6. Analysis of Total Phenolic Content

The total phenolic content was determined according to the Folin-Ciocalteau procedure [199]. In brief, transferring the extracts (100 μL) into test tubes filled to the top with ultrapure water (500 μL total tube volume) was followed by oxidation of the mixtures with 250 μL Folin-Ciocalteau reagent and then neutralization of the mixtures with a 20% aqueous Na_2_CO_3_ solution (1.25 mL) in a 5-min reaction. After the appearance of a distinctive blue colour in 40 min at room temperature, the mixtures were centrifuged at 4000× *g* for 5 min. The absorbance of the clear supernatants was measured at 725 nm against a blank containing an extraction solvent system instead of a sample. The total phenolic content of each sample was determined according to a calibration curve that was prepared with gallic acid, and the results are expressed as µg of gallic acid equivalent (GAE) per g of dry matter.

#### 4.3.7. Analysis of Phenolic Acids

Chromatographic analyses of the extracts for phenolic acids (PA) were performed on a Thermo Scientific Ultimate 3000 HPLC system equipped with a photodiode array detector, a quaternary pump, an autosampler, a column oven, and a refractive index detector (RID). The PA were separated on the Thermo Scientific Hypersil GOLD aQ^®^ C18 column (150 mm × 4.6 mm, i.d., 3 µm) using a linear gradient elution programme with a mobile phase containing the solvent A (formic acid/H_2_O, 1:99, *v*/*v*) and the solvent B (pure methanol) at a flow rate of 0.8 mL/min, with the following gradient profile: linear gradient elution from 10 to 60% B, 0–15 min; isocratic elution of 60% B, 15–20 min; linear gradient elution from 60 to 10% B, 20–25 min; and isocratic elution of 10% B, 25–30 min. The chromatograms were recorded at 280 nm by monitoring spectra within the 190–400 nm wavelength range [200]. Identified phenolic acids were confirmed and quantified by data acquisition and spectral evaluation using the Thermo Scientific Dionex Chromeleon software package (version 7.2). This involved a comparison of PA retention times with those of corresponding standards and spiking the samples with the appropriate standard. The PA content was expressed as μg per g of dry matter.

#### 4.3.8. Analysis of Tocopherols and Carotenoids

The extraction of tocopherols (*α*-T, *β+γ*-T, and *δ*-T) was done according to the slightly modified method of Gliszczyńska-Swigło and Sikorska [201]. After homogenisation of 0.2 g of maize flour with 4 mL of 2-propanol in the ultrasound bath at 25 °C for 30 min, the extracts were centrifuged at 15,000× *g* for 5 min, filtered through a 0.45 μm nylon syringe filter (Thermo Scientific, Dreieich, Germany), and directly injected into the Dionex UltiMate 3000 HPLC system (Thermo Scientific, Germany). The analytical column Hypersil GOLD aQ^®^ C18 (150 mm × 3 mm, 3 μm) was exploited for tocopherols separation, and the mixture of acetonitrile and methanol (1:1, *v*/*v*) at isocratic program, 1 mL/min, served as the mobile phase for the tocopherols separation.

The extraction of carotenoids (lutein+zeaxanthin (L+Z), and β-carotene) was performed by the slightly modified method of Rivera and Canela [202]. A mixture of methanol and ethyl acetate (6:4, *v*/*v*) was used as the solvent system for carotenoids extraction from 2 g of maize flour. The extraction was replicated two times (2 × 5 mL) in the ultrasound bath at 25 °C for 30 min, after which the extracts were centrifuged at 15,000× *g* for 5 min and filtered through a 0.45 μm nylon syringe filter. Prior to injection into the Dionex UltiMate 3000 HPLC system (Thermo Scientific, Germany), the extracts were evaporated to dryness under a nitrogen stream and re-dissolved in the mobile phase. For carotenoids separation, the analytical column Hypersil GOLD aQ^®^ C18 (150 mm × 4.6 mm, 3 μm) was employed, and the mixture of methanol and acetonitrile (90:10, *v*/*v*) at isocratic program, 1 mL/min, served as the mobile phase for the carotenoids separation [203].

The detection of tocopherols and carotenoids was conducted by fluorescence (λem of 325 nm; λex at 290 nm). The identification of tocopherols and carotenoids was realized by comparing their retention times with those of corresponding standards and by spiking the samples with the appropriate standard. For additional identification of the compounds on the basis of their absorption spectra, a photodiode-array detector set at 450 nm for tocopherols and 470 nm for carotenoids, respectively, was exploited.

The content of the analysed phytochemicals is expressed as μg per g of dry matter.

#### 4.3.9. Analysis of Total Antioxidant Capacity

The total antioxidant capacity (TAC) of maize flour obtained from the evaluated maize seed lot samples was determined according to the QUENCHER method described by Serpen et al. [136] using the 2,2-azino-bis/3-ethylbenzothiazoline-6-sulfonic acid (ABTS) reagent. Maize flour (10 mg) was mixed with 20 mL of ABTS working solution, and the mixture was intensively shaken for 25 min. After centrifugation at 9200× *g* for 5 min, the optically clear supernatant was separated, and the absorbance measurement was performed at 734 nm on a spectrophotometer (Agilent 8453 UV-visible Spectroscopy System, Agilent, Santa Clara, CA, USA). The antioxidant capacity was expressed as Trolox equivalent antioxidant capacity (TEAC) in µmol of Trolox per g of dry matter.

#### 4.3.10. Statistical Analysis

The results were statistically analysed using a two-way factorial analysis of variance (ANOVA) for a randomized complete block design (RCBD) and presented as the mean ± standard deviation (SD). For each evaluated parameter, the coefficient of variation (CV) was calculated. Significant differences between the accession means were determined by Fisher’s least significant difference (LSD) test at the 0.05 probability level, and differences with *p* ≤ 0.05 were considered significant. SPSS software for Windows, version 14.0 (SPSS Inc., Chicago, IL, USA), was used for the statistical analyses.

Pearson’s correlations were used to compare the performance of naturally aged seeds under cold storage (CS) and freshly harvested seeds (i.e., matching controls) in order to ascertain the relationship between seed vigour and germinability—important indicators of seed performance in the field, i.e., seedling growth parameters and measured biochemical markers.

## 5. Conclusions

Seed ageing is a complex biological trait involving an intricate network of molecular, biochemical, physiological, and metabolic processes, making it challenging to monitor. By comparing aged seeds with non-aged controls, we successfully identified physiological and biochemical markers—referred to as “signals”—that reliably indicated the degree of seed deterioration caused by natural ageing. It has been determined that, during natural ageing under medium-term cold storage conditions, the quality of proteins and their post-translational modifications—rather than their quantity—determine the consequences of seed ageing. In addition, changes in sugar content, particularly fructose and sucrose, and their relationship with early seedling growth suggest that sugar dynamics could serve as valuable biochemical markers for assessing seed physiological quality, ageing status, and seed stress history. The findings also highlight the dual influence of IBPs and FPs on seed germination and early seedling development, with these effects being modulated by seed age, stress history, and genetic factors. Moreover, correlation analysis indicated that overall seedling performance is strongly affected by the specific form of these compounds—whether insoluble-bound or soluble-free. Ageing-related oxidative stress from prolonged cold storage led to a consistent net decline in tocopherol isomers, while water deficit-induced oxidative stress during the post-zygotic phase caused a significant increase in the *α*-tocopherol isomer. Both forms of oxidative stress are the primary factors behind the reduction in *β*-carotene and lutein+zeaxanthin contents, which consistently decrease in parallel with seed ageing. Similar TAC values observed in CS1 and CS2 seed samples, comparable to non-aged controls, may indicate a state of elevated oxidative stress that results in increased antioxidant consumption. Our findings provide a solid foundation for future research to link the evaluated markers to enzyme activity and hormonal regulation governing and controlling seed ageing. We anticipate that, once fully identified, these complex ageing signals will be more extensively used as relevant biomarkers to enhance seed viability monitoring and advance ex situ conservation efforts.

## Figures and Tables

**Table 1:** Seedling growth parameters of evaluated maize seed lot samples.

Sample	Root FW		Shoot FW		Seed Rest FW		Root DW		Shoot DW		Seed Rest DW		Root Length		Shoot Length	
	g												cm			
1	0.051 ± 0.20	c	0.076 ± 0.04	d	0.254 ± 0.12	c	0.005 ± 0.00	cd	0.007 ± 0.00	bcde	0.136 ± 0.06	de	5.83 ± 0.29	d	3.233 ± 0.23	d
2	0.267 ± 0.03	a	0.312 ± 0.011	ab	0.449 ± 0.19	b	0.023 ± 0.01	ab	0.025 ± 0.01	a	0.208 ± 0.09	cd	14.67 ± 0.12	a	7.133 ± 0.21	a
3	0.289 ± 0.00	ab	0.349 ± 0.14	a	0.540 ± 0.25	ab	0.026 ± 0.01	a	0.030 ± 0.01	a	0.243 ± 0.11	bc	14.83 ± 1.28	a	7.467 ± 0.29	a
4	0.078 ± 0.01	c	0.065 ± 0.03	d	0.241 ± 0.10	c	0.006 ± 0.00	bcd	0.007 ± 0.00	cde	0.137 ± 0.06	de	7.10 ± 0.35	d	2.400 ± 0.34	e
5	0.289 ± 0.04	ab	0.184 ± 0.08	c	0.536 ± 0.23	ab	0.023 ± 0.01	ab	0.018 ± 0.01	abcde	0.294 ± 0.12	ab	11.47 ± 0.91	c	4.133 ± 0.16	c
6	0.369 ± 0.01	a	0.227 ± 0.11	c	0.566 ± 0.24	ab	0.032 ± 0.02	a	0.024 ± 0.01	ab	0.311 ± 0.13	ab	11.77 ± 0.32	bc	4.033 ± 0.28	c
7	0.028 ± 0.04	c	0.031 ± 0.01	d	0.201 ± 0.08	c	0.003 ± 0.00	d	0.003 ± 0.00	e	0.117 ± 0.04	e	4.30 ± 0.29	e	1.667 ± 0.14	f
8	0.225 ± 0.08	b	0.212 ± 0.10	c	0.553 ± 0.25	ab	0.020 ± 0.01	abc	0.021 ± 0.01	abcd	0.311 ± 0.14	ab	11.83 ± 1.03	bc	4.067 ± 0.22	c
9	0.206 ± 0.11	b	0.221 ± 0.10	c	0.574 ± 0.29	a	0.020 ± 0.01	abc	0.023 ± 0.01	abc	0.329 ± 0.17	a	12.47 ± 1.16	bc	4.167 ± 0.05	c
10	0.043 ± 0.04	c	0.063 ± 0.09	d	0.237 ± 0.10	c	0.004 ± 0.00	cd	0.006 ± 0.00	de	0.132 ± 0.05	de	6.68 ± 0.58	d	2.867 ± 0.15	d
11	0.231 ± 0.11	b	0.229 ± 0.09	c	0.461 ± 0.20	ab	0.020 ± 0.01	abc	0.022 ± 0.01	abcd	0.248 ± 0.11	bc	12.57 ± 0.94	bc	4.800 ± 0.42	b
12	0.266 ± 0.04	b	0.249 ± 0.12	bc	0.545 ± 0.25	ab	0.025 ± 0.01	a	0.023 ± 0.01	a	0.298 ± 0.13	ab	13.07 ± 1.34	b	5.167 ± 0.29	b
CV (%)	28.15		23.20		17.05		27.90		22.87		17.33		7.68		6.05	
LSD_0.05_	0.0928		0.0757		0.1197		0.0169		0.0169		0.0757		1.371		0.4383	

Legend: FW—fresh weight; DW—dry weight; CV—coefficient of variation; LSD—least significant difference. The results are mean ± SD of triplicate measurements (*n* = 3). Means followed by the same letter within the same column are not significantly different (*p* ≤ 0.05). Samples 1, 4, 7, and 10—the original, CS1 samples (harvested in 1985); samples 2, 5, 8, and 11—CS2 samples (regenerated in 2017); samples 3, 6, 9, and 12—the control samples (freshly regenerated in 2022). The seed samples belong to the following maize genotypes: MRIZP13509 (samples 1, 2, and 3); MRIZP13695 (samples 4, 5, and 6); MRIZP13687 (samples 7, 8, and 9); MRIZP13706 (samples 10, 11, and 12).

**Table 2:** The total protein and protein fractions content in the evaluated maize seed lot samples.

Sample	Total Protein		Albumins		Globulins		Glutelins		α-Zeins	
	% d.m.									
1	12.90 ± 0.20	b	0.765 ± 0.02	d	1.225 ± 0.02	e	2.915 ± 0.02	b	2.915 ± 0.02	f
2	13.54 ± 0.03	a	0.655 ± 0.04	ef	1.255 ± 0.02	d	3.035 ± 0.05	a	2.815 ± 0.02	g
3	12.93 ± 0.00	b	1.090 ± 0.03	a	1.255 ± 0.01	d	2.755 ± 0.06	c	3.370 ± 0.03	c
4	11.79 ± 0.01	fg	0.515 ± 0.02	g	1.125 ± 0.01	i	2.420 ± 0.03	de	3.060 ± 0.06	e
5	12.38 ± 0.04	d	0.710 ± 0.03	de	1.085 ± 0.02	j	2.075 ± 0.04	h	3.270 ± 0.00	d
6	11.93 ± 0.01	ef	0.740 ± 0.01	d	1.150 ± 0.01	g	2.335 ± 0.02	ef	3.300 ± 0.01	d
7	11.70 ± 0.04	g	0.425 ± 0.04	h	1.280 ± 0.01	a	2.310 ± 0.01	f	2.860 ± 0.03	fg
8	12.06 ± 0.08	e	0.620 ± 0.01	f	1.260 ± 0.01	c	2.260 ± 0.03	fg	2.740 ± 0.06	h
9	12.37 ± 0.11	d	0.650 ± 0.03	ef	1.265 ± 0.01	b	2.440 ± 0.03	d	3.100 ± 0.01	e
10	12.04 ± 0.04	e	0.365 ± 0.04	h	1.015 ± 0.05	k	2.295 ± 0.06	fg	3.650 ± 0.01	b
11	12.80 ± 0.11	bc	0.930 ± 0.01	b	1.155 ± 0.02	f	1.885 ± 0.06	i	3.760 ± 0.04	a
12	12.65 ± 0.04	c	0.855 ± 0.01	c	1.140 ± 0.01	h	2.205 ± 0.08	g	3.670 ± 0.01	b
CV (%)	0.64		3.80		1.84		1.99		0.96	
LSD_0.05_	0.1705		0.0696		0.0022		0.0984		0.0696	

Legend: d.m.—dry matter; CV—coefficient of variation; LSD—least significant difference. The results are mean ± SD of triplicate measurements (*n* = 3). Means followed by the same letter within the same column are not significantly different (*p* ≤ 0.05). Samples 1, 4, 7, and 10—the original, CS1 samples (harvested in 1985); samples 2, 5, 8, and 11—CS2 samples (regenerated in 2017); samples 3, 6, 9, and 12—the control samples (freshly regenerated in 2022). The seed samples belong to the following maize genotypes: MRIZP13509 (samples 1, 2, and 3); MRIZP13695 (samples 4, 5, and 6); MRIZP13687 (samples 7, 8, and 9); MRIZP13706 (samples 10, 11, and 12).

**Table 3:** The reducing and non-reducing sugars content in evaluated maize seed lot samples.

Sample	Glucose		Fructose		Sucrose		Maltose	
	% d.m.							
1	0.467 ± 0.001	h	0.188 ± 0.00	h	1.52 ± 0.04	a	1.089 ± 0.01	bcd
2	0.436 ± 0.00	i	0.231 ± 0.00	g	1.322 ± 0.02	bcd	0.856 ± 0.00	efg
3	0.692 ± 0.002	b	0.366 ± 0.01	c	1.072 ± 0.01	g	1.277 ± 0.02	ab
4	0.522 ± 0.00	g	0.269 ± 0.00	e	1.173 ± 0.01	efg	0.842 ± 0.00	efg
5	0.644 ± 0.01	d	0.288 ± 0.00	d	1.343 ± 0.00	bc	1.113 ± 0.01	bc
6	0.697 ± 0.01	b	0.230 ± 0.00	g	1.203 ± 0.02	def	1.441 ± 0.34	a
7	0.656 ± 0.00	c	0.395 ± 0.00	b	1.088 ± 0.18	fg	0.671 ± 0.00	g
8	0.586 ± 0.00	e	0.250 ± 0.00	f	1.238 ± 0.01	cde	0.889 ± 0.01	defg
9	0.658 ± 0.05	c	0.184 ± 0.01	h	1.216 ± 0.01	de	0.956 ± 0.05	cdef
10	0.713 ± 0.00	a	0.439 ± 0.00	a	1.172 ± 0.00	fg	0.762 ± 0.01	fg
11	0.559 ± 0.00	f	0.182 ± 0.00	h	1.421 ± 0.00	ab	1.022 ± 0.00	cde
12	0.641 ± 0.02	d	0.253 ± 0.01	f	1.247 ± 0.02	cde	0.915 ± 0.03	cdef
CV (%)	2.77		1.65		4.41		10.25	
LSD_0.05_	0.00696		0.0070		0.06028		0.2201	

Legend: d.m.—dry matter; CV—coefficient of variation; LSD—least significant difference. The results are mean ± SD of triplicate measurements (*n* = 3). Means followed by the same letter within the same column are not significantly different (*p* ≤ 0.05). Samples 1, 4, 7, and 10—the original, CS1 samples (harvested in 1985); samples 2, 5, 8, and 11—CS2 samples (regenerated in 2017); samples 3, 6, 9, and 12—the control samples (freshly regenerated in 2022). The seed samples belong to the following maize genotypes: MRIZP13509 (samples 1, 2, and 3); MRIZP13695 (samples 4, 5, and 6); MRIZP13687 (samples 7, 8, and 9); MRIZP13706 (samples 10, 11, and 12).

**Table 4:** (**a**) The total soluble-free phenolic compounds and detected soluble-free phenolic acids content in evaluated maize seed lot samples. (**b**) The total insoluble-bound phenolic compounds and detected insoluble-bound phenolic acids content in evaluated maize seed lot samples.

(a)								
Sample	Total FPs		Free *p*-CouA		Free FA		Free CA	
	μg GAE g^−1^ d.m.		μg g^−1^ d.m.					
1	219.71 ± 15.1	e	3.73 ± 0.07	d	1.69 ± 0.22	bc	n.d. *	
2	213.48 ± 15.2	e	2.00 ± 0.30	g	1.20 ± 0.04	cdef	n.d.	
3	211.37 ± 22.5	e	1.56 ± 0.30	h	1.04 ± 0.09	defg	n.d.	
4	319.28 ± 34.7	b	4.49 ± 0.00	bc	2.18 ± 0.04	b	2.56 ± 0.13	b
5	353.81 ± 28.7	a	4.40 ± 0.04	c	1.29 ± 0.17	cde	4.62 ± 0.26	ab
6	315.55 ± 18.0	b	3.75 ± 0.13	d	1.25 ± 0.22	cde	3.51 ± 0.49	ab
7	248.60 ± 12.1	cd	4.63 ± 0.09	bc	1.30 ± 0.17	cde	n.d.	
8	247.22 ± 19.7	d	3.21 ± 0.17	e	0.68 ± 0.09	fg	n.d.	
9	218.68 ± 19.4	e	4.69 ± 0.09	b	0.64 ± 0.04	g	3.83 ± 0.26	ab
10	259.75 ± 13.6	cd	5.05 ± 0.17	a	3.54 ± 0.04	a	n.d.	
11	268.32 ± 39.2	c	2.94 ± 0.17	f	1.56 ± 0.30	cd	3.86 ± 0.26	ab
12	267.45 ± 32.8	c	3.18 ± 0.21	e	0.76 ± 0.13	efg	5.34 ± 0.26	a
CV (%)	3.46		2.95		7.47		18.49	
LSD_0.05_	19.95		0.2308		0.5480		2.544	
**(b)**								
**Sample**	**total IBPs**		**bound *p*-CouA**		**bound FA**		**bound CA**	
	**μg GAE g^−1^ d.m.**		**μg g^−1^ d.m.**					
1	2454.3 ± 138.2	bc	331.26 ± 3.64	b	2412.32 ± 11.81	d	1.88 ± 0.08	d
2	2744.4 ± 136.3	a	359.84 ± 6.86	a	2655.54 ± 6.45	b	2.53 ± 0.13	a
3	2486.3 ± 85.6	b	287.06 ± 6.47	c	2370.91 ± 1.09	e	2.56 ± 0.19	a
4	2340.9 ± 71.1	bcd	188.61 ± 6.90	g	2307.14 ± 10.84	f	1.54 ± 0.02	e
5	2446.5 ± 262.1	bc	260.48 ± 3.45	d	2746.20 ± 6.23	a	1.89 ± 0.08	d
6	2329.1 ± 185.5	cd	224.39 ± 5.41	f	2264.17 ± 2.80	g	1.62 ± 0.15	e
7	2157.9 ± 56.3	e	192.59 ± 10.44	g	2027.14 ± 12.84	i	1.89 ± 0.08	d
8	2200.3 ± 60.0	de	272.18 ± 3.35	cd	2115.23 ± 11.35	h	1.93 ± 0.14	cd
9	2169.3 ± 40.7	e	242.73 ± 10.74	e	1992.43 ± 4.01	j	2.11 ± 0.00	bc
10	2454.4 ± 26.2	bc	193.29 ± 8.76	g	2478.78 ± 11.78	c	2.17 ± 0.04	b
11	2278.7 ± 97.0	de	197.28 ± 6.14	g	2260.00 ± 16.48	g	1.64 ± 0.17	e
12	2245.6 ± 121.7	de	230.90 ± 2.89	ef	2120.10 ± 22.07	h	1.89 ± 0.13	d
CV (%)	2.85		2.79		0.40		4.53	
LSD_0.05_	148.1		7.622		20.25		0.1969	

(**a**) Legend: FPs—soluble-free phenolics; *p*-CouA—*p*-coumaric acid; FA—ferulic acid; CA—caffeic acid; GAE—gallic acid equivalent; d.m.—dry matter; CV—coefficient of variation; LSD—least significant difference. The results are mean ± SD of triplicate measurements (*n* = 3). Means followed by the same letter within the same column are not significantly different (*p* ≤ 0.05); * n.d.—not detected. Samples 1, 4, 7, and 10—the original, CS1 samples (harvested in 1985); samples 2, 5, 8, and 11—CS2 samples (regenerated in 2017); samples 3, 6, 9, and 12—the control samples (freshly regenerated in 2022). The seed samples belong to the following maize genotypes: MRIZP13509 (samples 1, 2, and 3); MRIZP13695 (samples 4, 5, and 6); MRIZP13687 (samples 7, 8, and 9); MRIZP13706 (samples 10, 11, and 12). (**b**) Legend: IBPs—insoluble-bound phenolics; *p*-CouA—*p*-coumaric acid; FA—ferulic acid; CA—caffeic acid; GAE—gallic acid equivalent; d.m.—dry matter; CV—coefficient of variation; LSD—least significant difference. The results are mean ± SD of triplicate measurements (*n* = 3). Means followed by the same letter within the same column are not significantly different (*p* ≤ 0.05). Samples 1, 4, 7, and 10—the original, CS1 samples (harvested in 1985); samples 2, 5, 8, and 11—CS2 samples (regenerated in 2017); samples 3, 6, 9, and 12—the control samples (freshly regenerated in 2022). The seed samples belong to the following maize genotypes: MRIZP13509 (samples 1, 2, and 3); MRIZP13695 (samples 4, 5, and 6); MRIZP13687 (samples 7, 8, and 9); MRIZP13706 (samples 10, 11, and 12).

**Table 5:** The tocopherols and carotenoids content in evaluated maize seed lot samples.

Sample	*α*-T		*β+γ*-T		*δ*-T		*β*-Carotene		Lut+Zeaxanth	
	μg g^−1^ d.m.									
1	8.23 ± 0.46	e	10.18 ± 0.57	j	0.59 ± 0.03	g	0.35 ± 0.00	i	9.06 ± 0.12	h
2	11.48 ± 0.49	b	18.92 ± 0.81	i	1.10 ± 0.04	f	1.92 ± 0.06	h	16.64 ± 0.34	f
3	10.76 ± 0.42	c	22.38 ± 0.87	h	1.40 ± 0.05	ef	2.61 ± 0.07	g	21.52 ± 0.43	d
4	4.24 ± 0.21	j	39.93 ± 1.94	ef	0.43 ± 0.12	g	1.79 ± 0.05	h	4.97 ± 0.10	k
5	6.71 ± 0.28	h	48.19 ± 2.02	c	1.42 ± 0.10	e	9.78 ± 0.29	d	14.25 ± 0.23	g
6	4.29 ± 0.21	j	41.63 ± 2.17	e	2.02 ± 0.10	bc	16.87 ± 0.49	a	22.61 ± 0.43	c
7	5.25 ± 0.23	i	32.11 ± 1.46	g	1.59 ± 0.08	de	2.91 ± 0.08	g	6.43 ± 0.13	i
8	8.87 ± 0.38	d	39.36 ± 1.69	f	1.80 ± 0.06	cd	8.41 ± 0.25	f	17.41 ± 0.45	e
9	12.12 ± 0.54	a	39.40 ± 1.75	f	1.44 ± 0.06	e	15.58 ± 0.45	b	35.44 ± 0.54	a
10	5.09 ± 0.26	i	45.68 ± 2.33	d	2.32 ± 0.12	b	0.33 ± 0.04	i	5.83 ± 0.13	j
11	7.42 ± 0.40	f	54.64 ± 2.91	b	2.96 ± 0.16	a	8.81 ± 0.25	e	16.69 ± 0.29	f
12	7.12 ± 0.37	g	58.90 ± 3.12	a	2.86 ± 0.15	a	14.33 ± 0.42	c	32.01 ± 0.51	b
CV (%)	1.49		2.10		2.47		2.59		0.97	
LSD_0.05_	0.2510		1.737		0.1797		0.6603		0.3617	

Legend: T—tochopherols; d.m.—dry matter; CV—coefficient of variation; LSD—least significant difference. The results are mean ± SD of triplicate measurements (*n* = 3). Means followed by the same letter within the same column are not significantly different (*p* ≤ 0.05). Samples 1, 4, 7, and 10—the original, CS1 samples (harvested in 1985); samples 2, 5, 8, and 11—CS2 samples (regenerated in 2017); samples 3, 6, 9, and 12—the control samples (freshly regenerated in 2022). The seed samples belong to the following maize genotypes: MRIZP13509 (samples 1, 2, and 3); MRIZP13695 (samples 4, 5, and 6); MRIZP13687 (samples 7, 8, and 9); MRIZP13706 (samples 10, 11, and 12).

**Table 6:** Pearson’s correlations between biochemical markers and seedling growth parameters of evaluated maize seed lot samples differing in the period of maintenance under cold storage.

	Root Length			Shoot Length			SVI-I			SVI-II		
	Control	CS1	CS2	Control	CS1	CS2	Control	CS1	CS2	Control	CS1	CS2
total protein	0.712	0.076	0.823	0.859	0.796	0.945	0.774	−0.091	0.875	−0.883	−0.199	−0.905
glutelins	0.558	0.017	0.726	0.743	0.639	0.852	0.633	−0.045	0.823	−0.713	−0.077	−0.501
fructose	0.705	−0.186	−0.359	0.953	−0.446	−0.301	0.887	−0.217	−0.275	−0.878	−0.208	0.592
sucrose	−0.640	0.086	0.108	−0.834	0.694	0.117	−0.776	−0.021	0.061	0.771	−0.077	−0.507
total FPs	−0.453	0.551	−0.667	−0.576	−0.335	−0.590	−0.482	0.649	−0.639	0.521	0.643	0.420
free *p*-CouA	−0.701	0.095	−0.793	−0.918	−0.393	−0.820	−0.856	0.095	−0.798	0.889	0.080	0.733
total IBPs	0.581	0.645	0.559	0.640	0.798	0.817	0.632	0.447	0.695	−0.677	0.325	−0.688
bound *p*-CouA	0.709	−0.072	0.604	0.899	0.643	0.718	0.825	−0.191	0.711	−0.829	−0.243	−0.307
bound FA	0.394	0.784	0.240	0.674	0.890	0.402	0.602	0.541	0.346	−0.569	0.351	−0.284
bound CA	0.704	−0.230	0.680	0.820	0.245	0.774	0.771	−0.437	0.773	−0.799	−0.522	−0.396
*β+γ*-tocopherols	−0.408	0.361	−0.696	−0.594	−0.354	−0.784	−0.501	0.380	−0.783	0.529	0.347	0.398
*β*-carotene	−0.767	−0.551	−0.848	−0.971	−0.925	−0.942	−0.896	−0.323	−0.916	0.930	−0.150	0.686

Legend: SVI—Seedling Vigour Index; FPs—total soluble-free phenolic compounds; IBPs—total insoluble-bound phenolic compounds; *p*-CouA—*p*-coumaric acid; FA—ferulic acid; CA—caffeic acid. The critical values in a two-tailed test for a number of data (*n* = 12) are as follows: ≥0.576 (at significance level *α* = 0.05), ≥0.708 (*α* = 0.01), and ≥0.823 (*α* = 0.001), respectively. The colour intensity reflects the level of changes during natural seed-ageing. The colour distinctions (pink vs. blue) emphasizes the specific roles that certain biochemical markers play in the ageing process.

**Table 7:** Passport data for the examined MRIZP gene bank maize accessions.

Sample	ID No.	Accession Name	Country of Origin	Year of the Last Regeneration
1	MRIZP13509	Illinois composite	Mexico	1985
2	MRIZP13509	Illinois composite	Mexico	2017
3	MRIZP13509	Illinois composite	Mexico	2022
4	MRIZP13695	BK × ETO-2007 × 00	Argentina	1985
5	MRIZP13695	BK × ETO-2007 × 00	Argentina	2017
6	MRIZP13695	BK × ETO-2007 × 00	Argentina	2022
7	MRIZP13687	BK × ETO-2004 × 08	Argentina	1985
8	MRIZP13687	BK × ETO-2004 × 08	Argentina	2017
9	MRIZP13687	BK × ETO-2004 × 08	Argentina	2022
10	MRIZP13706	BK × ETO-1995 × 07	Argentina	1985
11	MRIZP13706	BK × ETO-1995 × 07	Argentina	2017
12	MRIZP13706	BK × ETO-1995 × 07	Argentina	2022

Legend: MRIZP—Maize Research Institute Zemun Polje. Samples 1, 4, 7, and 10—the original, CS1 samples (harvested in 1985); samples 2, 5, 8, and 11—CS2 samples (regenerated in 2017); samples 3, 6, 9, and 12—the control samples (freshly regenerated in 2022).

## Data Availability

The original contributions presented in this study are included in the article. Further inquiries can be directed to the corresponding author.

## Abbreviations

The following abbreviations are used in this manuscript:

ROS	Reactive Oxygen Species
MRIZP	Maize Research Institute Zemun Polje
SVI	Seedling Vigour Index
GAE	Gallic Acid Equivalent
TAC	Total Antioxidant Capacity
SSPs	Seed Storage Proteins
RNS	Reactive Nitrogen Species
ISTA	International Seed Testing Association
HPLC	High-Performance Liquid Chromatography
